# Augmented IFN-γ and TNF-α Induced by Probiotic Bacteria in NK Cells Mediate Differentiation of Stem-Like Tumors Leading to Inhibition of Tumor Growth and Reduction in Inflammatory Cytokine Release; Regulation by IL-10

**DOI:** 10.3389/fimmu.2015.00576

**Published:** 2015-12-02

**Authors:** Vickie T. Bui, Han-Ching Tseng, Anna Kozlowska, Phyu Ou Maung, Kawaljit Kaur, Paytsar Topchyan, Anahid Jewett

**Affiliations:** ^1^Division of Oral Biology and Oral Medicine, The Jane and Jerry Weintraub Center for Reconstructive Biotechnology, University of California Los Angeles, Los Angeles, CA, USA; ^2^The Jonsson Comprehensive Cancer Center, UCLA School of Dentistry and Medicine, Los Angeles, CA, USA; ^3^Department of Tumor Immunology, Poznan University of Medical Sciences, Poznan, Poland

**Keywords:** IFN-γ, NK cells, anti-IL10mAb, probiotic bacteria, monocytes, differentiation

## Abstract

Our previous reports demonstrated that the magnitude of natural killer (NK) cell-mediated cytotoxicity correlate directly with the stage and level of differentiation of tumor cells. In addition, we have shown previously that activated NK cells inhibit growth of cancer cells through induction of differentiation, resulting in the resistance of tumor cells to NK cell-mediated cytotoxicity through secreted cytokines, as well as direct NK-tumor cell contact. In this report, we show that in comparison to IL-2 + anti-CD16mAb-treated NK cells, activation of NK cells by probiotic bacteria (sAJ2) in combination with IL-2 and anti-CD16mAb substantially decreases tumor growth and induces maturation, differentiation, and resistance of oral squamous cancer stem cells, MIA PaCa-2 stem-like/poorly differentiated pancreatic tumors, and healthy stem cells of apical papillae through increased secretion of IFN-γ and TNF-α, as well as direct NK-tumor cell contact. Tumor resistance to NK cell-mediated killing induced by IL-2 + anti-CD16mAb + sAJ2-treated NK cells is induced by combination of IFN-γ and TNF-α since antibodies to both, and not each cytokine alone, were able to restore tumor sensitivity to NK cells. Increased surface expression of CD54, B7H1, and MHC-I on NK-differentiated tumors was mediated by IFN-γ since the addition of anti-IFN-γ abolished their increase and restored the ability of NK cells to trigger cytokine and chemokine release; whereas differentiated tumors inhibited cytokine release by the NK cells. Monocytes synergize with NK cells in the presence of probiotic bacteria to induce regulated differentiation of stem cells through secretion of IL-10 resulting in resistance to NK cell-mediated cytotoxicity and inhibition of cytokine release. Therefore, probiotic bacteria condition activated NK cells to provide augmented differentiation of cancer stem cells resulting in inhibition of tumor growth, and decreased inflammatory cytokine release.

## Introduction

Natural killer (NK) cells constitute 10–15% of human peripheral blood lymphocytes and are known as the first-line of defense against infections and neoplasia. Our recent studies indicate that NK cells select and differentiate both healthy and transformed stem-like cells, resulting in the maturation of target cells with which NK cells interact, and other cells in the surrounding tissues (1–4).

Probiotic bacteria were identified in the early twentieth century by Elie Metchnikoff, who found that certain strains of bacteria in the human gut were beneficial to homeostasis of the gut; therefore, these beneficial bacteria were named probiotics (5). They are commonly used in food and supplements to enhance innate immunity, such as NK cell activity, and to maintain microbial balance in the digestive tract (6–8). Majority of probiotics are lactic acid-producing bacteria, which includes *lactobacilli*, *streptococci*, and *bifidobacteria*. The study on probiotic bacteria is one of the fastest growing areas of research with promising outlook for use in the treatment of gut mucosal pathologies, obesity, metabolic syndrome, allergy, heart disease, and cancer prevention and treatment, to name a few (9–12). Probiotics have an effect on the production of immunoglobulin A (13–15), stimulation of macrophage activity (16), and are likely to lessen the toxicity effect of anti-cancer therapy (17). They induce differentiation of immature dendritic cells to regulatory dendritic cells, induce regulatory T cells, and increase the activity of NK cells, resulting in a local intestinal defense (5, 7). The wide range of benefits observed with the use of probiotic bacteria suggests their integral role in the modulation of local gut immunity, as well as systemic immunity (14, 18, 19).

In our previous study, we demonstrated that differentiation of healthy and transformed stem cells directly correlate with the cells’ sensitivity to NK cell lysis (20). In addition, we showed that NK cells have a significantly influential role in promoting cell differentiation by providing critical signals via secreted cytokines, as well as direct NK–stem cell interaction (2, 21–23).

Although a lot is known about the benefits and role of probiotic bacteria in immune modulation, the underlying mechanisms of such immune modulation by NK cells are not understood. In this paper, we provide potential mechanisms for the role of probiotic bacteria in regulating the function of NK cells in such a way that results in NK cell conditioning to support differentiation of the cells. In particular, probiotic bacteria induce significant split anergy in activated NK cells leading to decreased cytotoxicity, but significant induction of IFN-γ and TNF-α. Split anergy was coined previously in our laboratory to indicate decreased NK cell cytotoxicity in the presence of significant secretion of cytokines (4, 24, 25). Induction of split anergy in NK cells promotes differentiation of target cells, increases key differentiation receptors on tumor cells, induces tumor cell resistance to NK cell-mediated cytotoxicity, and inhibits inflammation due to a decrease or shut down of cytokine and chemokine release after tumor differentiation. In addition, probiotic bacteria induce significant expansion of NK cells (26).

## Materials and Methods

### Cell Lines, Reagents, and Antibodies

RPMI 1640 supplemented with 10% fetal bovine serum (FBS) (Gemini Bio-Products, CA, USA) was used for the cultures of human NK cells and monocytes. Oral squamous carcinoma stem cells (OSCSCs) were isolated from oral cancer patient with tongue tumors at UCLA and cultured in RPMI 1640 supplemented 10% FBS (Gemini Bio-Products, CA, USA), 1.4% antibiotic antimycotic, 1% sodium pyruvate, 1.4% non-essential amino acids, 1% l-glutamine, 0.2% gentamicin (Gemini Bio-Products, CA, USA), and 0.15% sodium bicarbonate (Fisher Scientific, PA, USA). MIA PaCa-2 (MP2) were cultured in DMEM with 10% FBS and 1% penicillin and streptomycin (Gemini Bio-Products, CA, USA). Stem cells from the apical papilla (SCAPs) were cultured in DMEM complete medium supplemented with 2% FBS and 1% penicillin and streptomycin (Gemini Bio-Products, CA, USA). Recombinant IL-2 was obtained from NIH-BRB. Antibodies to CD16 and IL-10 were purchased from Biolegend (San Diego, CA, USA). Anti-MHC-I was prepared in our laboratory, and 1:100 dilution was found to be the optimal concentration to use. PE-conjugated antibodies against CD44, CD54, CD69, CD107a, and B7H1 were obtained from Biolegend (San Diego, CA, USA). Flow cytometry analysis was performed using Beckman Coulter Epics XL cytometer (Brea, CA, USA). Antibodies to TNF-α and IFN-γ were prepared in our laboratory, and 1:100 dilution was found to be the optimal concentration to use for blocking experiments. Human NK and monocyte purification kits were obtained from Stem Cell Technologies (Vancouver, BC, Canada). Propidium iodide (PI) was purchased from Sigma Aldrich (Buffalo, NY, USA).

### Purification of Peripheral Blood NK Cells and Monocytes

PBMCs from healthy donors were isolated, and NK cells and monocytes were purified using isolation kits obtained from Stem Cell Technologies, as described before (27). The purity of NK cells and monocyte populations was found to be >90 and >95%, respectively, based on the flow cytometric analysis. Written informed consents approved by UCLA Institutional Review Board (IRB) were obtained from the blood donors, and all the procedures were approved by the UCLA IRB.

### ELISA and Multiplex Cytokine Array Kit

Single ELISA was performed as described previously (27). Fluorokine MAP cytokine multiplex kits were purchased from R&D Systems (Minneapolis, MN, USA), and the procedures were conducted as suggested by the manufacturer. Analysis was performed using the Star Station software.

### Surface Staining and Cell Death Assays

Staining was performed by labeling the cells with antibodies or PI, as described previously (27–29).

### ^51^Cr Release Cytotoxicity Assay

The ^51^Cr release assay was performed as described previously (30). Briefly, different numbers of purified NK cells were incubated with ^51^Cr-labeled target cells. After a 4-h incubation period, the supernatants were harvested from each sample and counted for released radioactivity using the gamma counter. The percentage specific cytotoxicity was calculated as follows:

$$\%\text{ cytotoxicity}=\frac{\text{experimental cpm}-\text{spontaneous cpm}}{\text{total cpm}-\text{spontaneous cpm}}$$

Lytic Units (LU) 30/10^6^ is calculated by using the inverse of the number of effector cells needed to lyse 30% of tumor target cells ×100.

### Bacteria Sonication

AJ2 is a combination of eight Gram-positive bacterial strains (*Streptococcus thermophilus*, *Bifidobacterium longum*, *Bifidobacterium breve*, *Bifidobacterium infantis*, *Lactobacillus acidophilus*, *Lactobacillus plantarum*, *Lactobacillus casei*, and *Lactobacillus bulgaricus*) used to induce differentiation of stem cells (4). AJ2 was re-suspended in RPMI supplemented with 10% FBS (Gemini Bio-Products) at a final concentration of 10^6^/mL. The sample was sonicated for 15 s while on ice, then incubated for 30 s on ice, and the sonication process was repeated 20 times to achieve complete sonication. Lastly, the sonicated samples were aliquoted and stored in −80°C freezer.

### Stem Cell Differentiation with NK Cell Supernatant

Human NK cells were purified from healthy donors’ PBMCs as described earlier. NK cells were left untreated or treated with a combination of IL-2 (1,000 U/mL) and anti-CD16mAb (3 μg/mL) with or without sonicated probiotic bacteria AJ2 (sAJ2) in the presence or absence of monocytes for 18 h before the supernatants were removed and used in differentiation experiments. The amounts of IFN-γ produced by activated NK cells were assessed with IFN-γ ELISA (Biolegend, CA, USA). Differentiation of OSCSCs was conducted with gradual daily addition of increasing amounts of NK cell supernatant. On average, a total of 4,500 pg of IFN-γ containing supernatants obtained from IL-2 + anti-CD16mAb + sAJ2-treated NK cells was added for 4 days to induce differentiation and resistance of OSCSCs to NK cell-mediated cytotoxicity. However, a total of 2,000 pg of IFN-γ containing supernatants obtained from IL-2 + anti-CD16mAb + sAJ2 + monocyte-treated NK cells was added for 3 days to induce differentiation and resistance of OSCSCs to NK cell-mediated cytotoxicity. SCAPs required on average a total of 1,400 pg of IFN-γ containing supernatants obtained from IL-2 + anti-CD16mAb + sAJ2-treated NK cells during a 4-day treatment, whereas MP2 tumors required a total of 7,000 pg of IFN-γ containing supernatants from IL-2 + anti-CD16mAb + sAJ2-treated NK cells for 4 days to promote differentiation and resistance to NK cell-mediated cytotoxicity. Afterwards, target cells were washed with 1× PBS, detached and used for experiments.

### Stem Cell Differentiation with 2% Paraformaldehyde Fixed NK Cells

Human NK cells were purified as described earlier. NK cells were left untreated or treated with a combination of IL-2 (1,000 U/mL) and anti-CD16mAb (3 μg/mL) and/or the presence of sAJ2 at 3:1 bacteria to NK cell ratio for 18 h. Afterwards, supernatants were removed, and the NK cells were fixed with freshly prepared 2% paraformaldehyde for 15 min. NK cells were then washed three times with 1× PBS and added to tumor cultures. Differentiation of OSCSCs was conducted on average for 5 days with daily and gradual addition of increasing amounts of fixed NK cells. During the differentiation process of OSCSCs, NK cells were added to tumor cells at an effector to target ratio of 0.6:1 for 4 days. For the differentiation of MP2, an effector to target ratio of 1.5:1 was added to culture for 4 days. After the treatment period, NK cells were removed from the tumors, and the target cells were used for experiments.

### Statistical Analysis

An unpaired, two-tailed Student’s *t*-test was performed for the statistical analysis. One way ANOVA with a Bonferroni post-test was used to compare the different groups.

## Results

### Induction of Differentiation and Resistance of OSCSCs and MP2 by Supernatants from Probiotic Bacteria and IL-2 + Anti-CD16mAb-Conditioned NK Cells were Mediated by IFN-γ and TNF-α

sAJ2 is a combination of eight strains of probiotic bacteria with the ability to induce synergistic production of IFN-γ when added to IL-2-treated or IL-2 + anti-CD16mAb-treated NK cells (Table S1 in Supplementary Material). The combination of strains was used to provide bacterial diversity in addition to synergistic induction of a balanced pro- and anti-inflammatory cytokine and growth factor release by untreated, IL-2-treated, or IL-2 + anti-CD16mAb-treated NK cells (Table S1 in Supplementary Material). Moreover, the quantity of each bacteria within the combination of strains was titrated and adjusted to yield a closer ratio of IFN-γ to IL-10 to that obtained when NK cells are activated with IL-2 + anti-CD16mAb in the absence of bacteria (Table S1 in Supplementary Material). The rationale behind such selection was to obtain a ratio similar to that obtained with NK cells activated with IL-2 + anti-CD16mAb in the absence of bacteria since such treatment provided significant differentiation of the cells (4). Interestingly, addition of IL-2 + anti-CD16mAb in the presence or absence of sAJ2 to NK cells had the highest induction of IFN-γ secretion when compared to untreated or IL-2 treated NK cells (Table S1 in Supplementary Material). No significant differences or a slight change in cytotoxicity could be seen either with sAJ2 or with each bacterial strain for untreated or IL-2-treated NK cell cytotoxicity (Figure S1 in Supplementary Material). Moreover, treatment of NK cells with IL-2 + anti-CD16mAb in the presence and absence of sAJ2 inhibited cytotoxicity when compared to IL-2-treated NK cells, and *B. longum* was able to reverse the inhibition of cytotoxicity moderately (Figure S1 in Supplementary Material). The data obtained by IL-2 + anti-CD16mAb-treated NK cells in the presence and absence of treatment with probiotic bacteria suggest dissociation of cytotoxicity and cytokine secretion for the effect of probiotic bacteria on NK cells since they trigger significant IFN-γ secretion in the presence of decreased NK cell-mediated cytotoxicity which we had previously coined as split anergy (Figure S1 and Table S1 in Supplementary Material).

**Figure 1:**
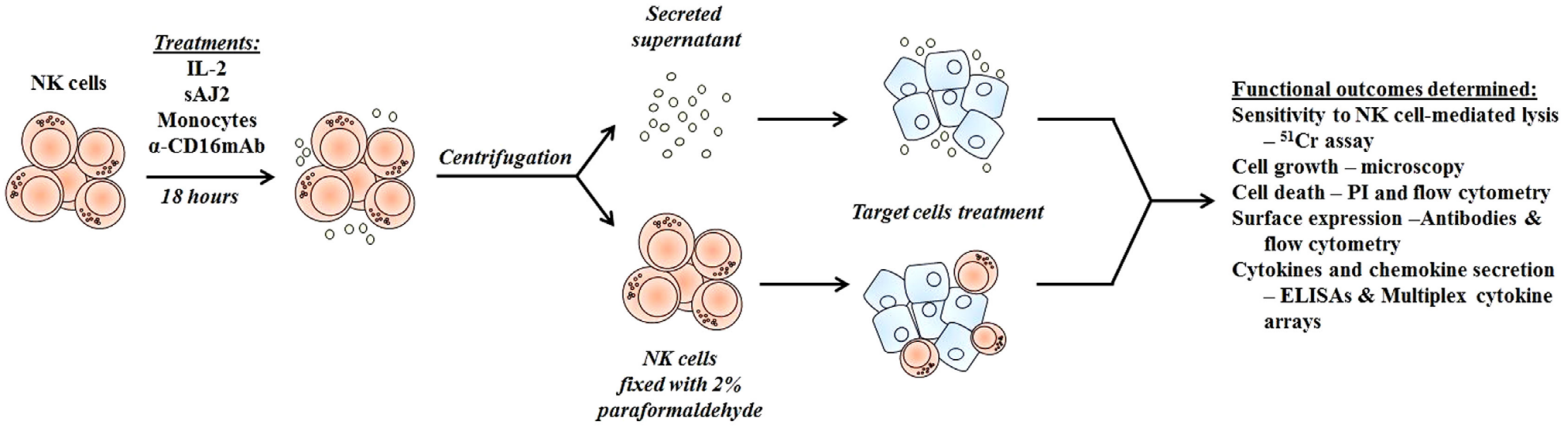
**Schematic representation of steps used to differentiate cancer stem-like cells with NK cell supernatants and fixed NK cells after their activation**. Highly purified NK cells were left untreated or treated with IL-2, sAJ2, monocytes, and/or anti-CD16mAb for 18 h, after which the treated NK cells were centrifuged, and the supernatants were removed and used to treat target cells. For cell–cell contact studies, treated NK cells were fixed with freshly prepared 2% paraformaldehyde for 15 min, washed three times with 1× PBS and added to target cell cultures. After the differentiation period, NK supernatants or fixed NK cells were removed from target cell cultures, and the targets were used in various assays.

**Figure 2:**
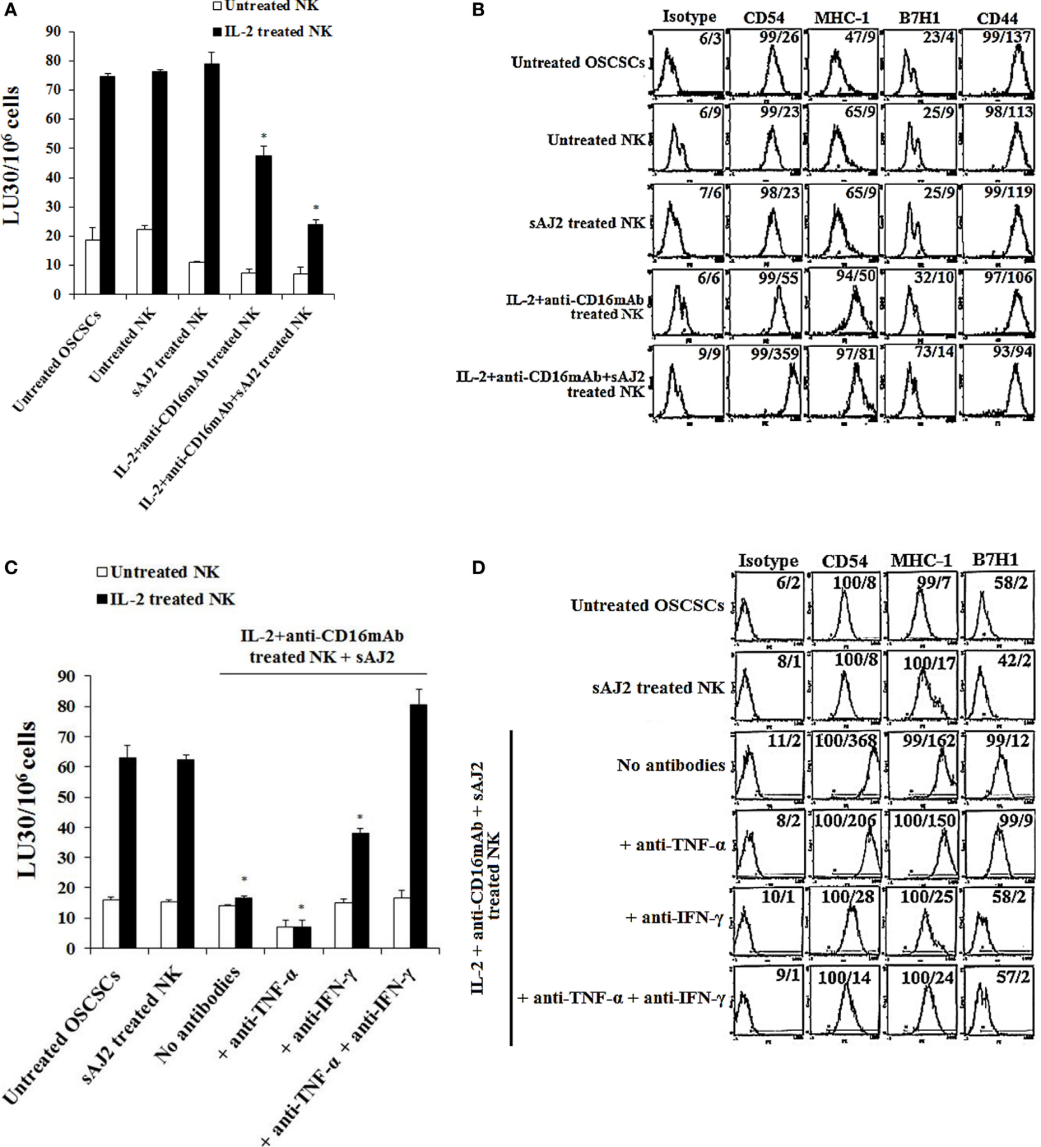

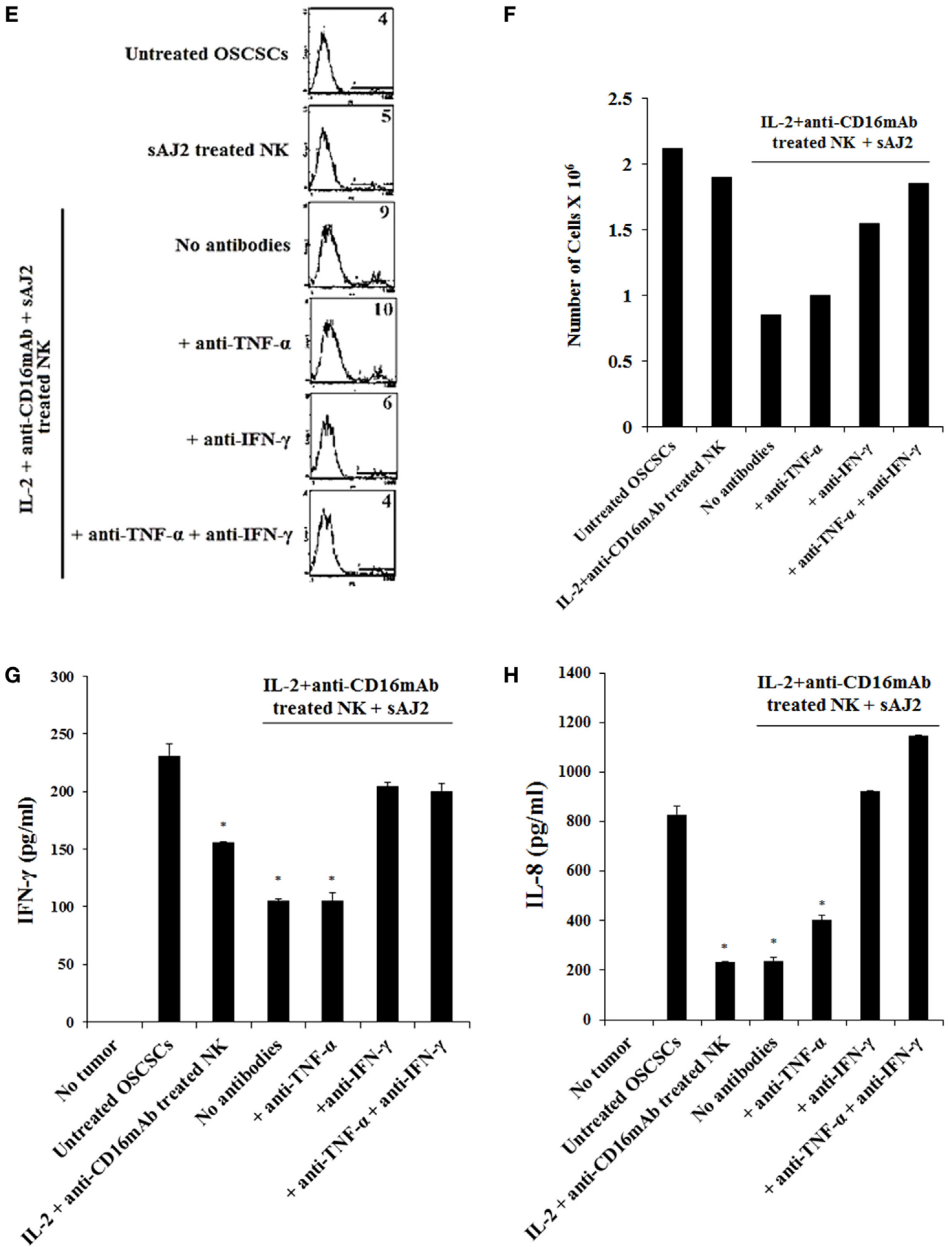
**Resistance to NK cell-mediated lysis of OSCSCs treated with supernatants from IL-2 + anti-CD16mAb + sAJ2-stimulated NK cells is mediated by the combination of IFN-γ and TNF-α and not each cytokine alone**. Highly purified NK cells were left untreated or treated with IL-2 (1,000 U/mL) and anti-CD16mAb (3 μg/mL) with or without sonicated AJ2 (sAJ2) at 1:3 (NK:sAJ2) ratio for 18 h before the supernatants were harvested and added to OSCSCs for 4 days. Afterwards, untreated OSCSCs and those treated with different NK cell supernatants indicated in the figure were detached from the tissue culture plates, extensively washed with 1× PBS and labeled with ^51^Cr. Freshly isolated NK cells were left untreated or treated with IL-2 (1,000 U/mL) for 24 h before the cells were used as effector cells in ^51^Cr release assay against OSCSCs treated with NK supernatants. The lytic units 30/10^6^ cells were determined using inverse number of NK cells required to lyse 30% of the target cells ×100. Differences between untreated OSCSCs and those cultured with IL-2 + anti-CD16mAb-treated NK or those stimulated with IL-2 + anti-CD16mAb + sAJ2-treated NKs were significant at a *P*-value of < 0.05 (*) **(A)**. OSCSCs were differentiated as described in **(A)**, and after 4 days the surface expression of CD54, MHC-1, B7H1, and CD44 on untreated OSCSCs or those treated with NK cell supernatants as shown in the figure was assessed after staining with the PE-conjugated antibodies and analyzed using flow cytometry. Isotype control antibodies were used as controls. The numbers on the right hand corner are the percentages and the mean channel fluorescence intensities for each histogram **(B)**. NK cells were treated with sAJ2 alone at 1:3 (NK:sAJ2) ratio or treated with IL-2 (1,000 U/mL) and anti-CD16mAb (3 μg/mL) and sAJ2 at 1:3 (NK:sAJ2) ratio in the presence of anti-TNF-α (1:100), anti-IFN-γ (1:100), or combination of anti-TNF-α (1:100) and anti-IFN-γ (1:100) as shown in the figure for 18 h. Afterwards, NK cell supernatants were harvested and added to OSCSCs for 4 days. Afterwards, untreated OSCSCs and those treated with NK cell supernatants were detached from the tissue culture plates, extensively washed with 1× PBS and labeled with ^51^Cr. Freshly isolated NK cells were left untreated or treated with IL-2 (1,000 U/mL) for 24 h before the cells were used as effector cells in ^51^Cr release assay against OSCSCs. The lytic units 30/10^6^ cells were determined using inverse number of NK cells required to lyse 30% of the target cells ×100. Differences between untreated OSCSCs and those stimulated with IL-2 + anti-CD16mAb + sAJ2-treated NK with or without anti-TNF-α or anti-IFN-γ were significant at a *P*-value of <0.05 (*) **(C)**. OSCSCs were treated with NK cell supernatants, as described in **(C)**, and after 4 days the surface expression of CD54, MHC-1, and B7H1 on untreated tumor cells or those treated with NK cell supernatants was assessed after staining with the PE-conjugated antibodies and analyzed using flow cytometry. Isotype control antibodies were used as controls. The numbers on the right hand corner are the percentages and the mean channel fluorescence intensities for each histogram **(D)**. OSCSCs were treated with NK cell supernatants as described in **(C)**, and the percent cell death in OSCSCs was determined using PI staining followed by flow cytometric analysis. The numbers on the right hand corner are the percentages of PI-positive cells for each histogram **(E)**. Purified NK cells were treated with a combination of IL-2 (1,000 U/mL) and anti-CD16mAb (3 μg/mL) in the presence or absence of sAJ2 at 1:3 (NK:sAJ2) ratio with or without anti-TNF-α (1:100) and/or anti-IFN-γ (1:100) for 18 h. Afterwards, supernatants were removed and used for the treatment of OSCSCs. OSCSCs (2 × 10^5^ cells/well) were cultured overnight before they were treated with supernatants obtained from the NK cells treated as indicated in the figure for 4 days. At the end of the incubation, OSCSCs were detached, and the numbers of cells were assessed using microscopy **(F)**. Freshly isolated NK cells were treated with IL-2 (1,000 U/mL) for 18 h. Afterwards, NK cells were added to untreated OSCSCs and those differentiated with NK cell supernatants, as described in **(C)**, at an effector to target ratio of 0.5–1. After an overnight incubation, the supernatants were removed from the cocultures, and the levels of IFN-γ **(G)** and IL-8 **(H)** secretions were determined using specific ELISAs. Differences between untreated OSCSCs and those stimulated with IL-2 + anti-CD16mAb-treated NK or stimulated with IL-2 + anti-CD16mAb-treated NK + sAJ2 with or without anti-TNF-α were significant at a *P*-value of <0.05 (*).

To determine whether supernatants obtained from probiotic bacteria and IL-2 + anti-CD16mAb-treated NK cells are capable of inducing differentiation and resistance in OSCSCs or in MP2 stem-like pancreatic tumors, NK cells were treated as described in (Figure 1), and the supernatants were removed and added to tumor cells. After differentiation, the susceptibility of tumor cells to NK cell-mediated lysis, the surface expression of CD54, MHC-1, B7H1, and CD44, and the induction of IFN-γ and IL-8 secretion by NK cells were assessed (Figure 2; Figure S2 in Supplementary Material). Treatment of OSCSCs (Figures 2A,C) and MP2 (Figure S2A in Supplementary Material) with supernatants from untreated NK cells or NK cells treated with sAJ2 did not cause significant differences in the susceptibility of OSCSCs or MP2 to IL-2-activated NK cell-mediated lysis (Figures 2A,C; Figure S2A in Supplementary Material). Supernatants obtained from IL-2 + anti-CD16mAb-treated NK cells mediated resistance in OSCSCs; however, the level of resistance to IL-2 activated NK cells was much more prominent in OSCSCs treated with supernatants obtained from IL-2 + anti-CD16mAb + sAJ2-treated NK cells (*P* < 0.05) (Figure 2A). We then studied the relationship between resistance of OSCSCs and MP2 to NK cell cytotoxicity and the modulation of key cell surface receptor expression on OSCSCs (Figures 2B,D) and MP2 (Figure S2B in Supplementary Material). Among many surface receptors tested, CD54, MHC-1, and B7H1 expressions on tumor cells were found to correlate significantly with the differentiation and resistance to NK cell-mediated lysis (4). NK cells treated with IL-2 + anti-CD16mAb + sAJ2 induced more differentiation when compared to OSCSCs (Figure 2B) treated with the supernatants obtained from IL-2 + anti-CD16mAb since significant increase in surface expression of CD54, MHC-1, and B7H1 and a moderate decrease in CD44 stem cell marker expression were observed on differentiated tumor cells. Similar results were obtained when MP2s were treated with supernatants from IL-2 + anti-CD16mAb + sAJ2-treated NK cells (Figure S2B in Supplementary Material). Supernatants from either untreated NK cells or NK cells treated with sAJ2 did not have any significant effects on the surface expression of OSCSCs (Figures 2B,D) and MP2 (Figure S2B in Supplementary Material). Indeed, the expression of CD107a and CD69 on IL-2 + anti-CD16mAb-treated NK cells was substantially higher in the presence of sAJ2 than in the absence of sAJ2 (Figure S3 in Supplementary Material).

To elucidate the mechanism by which OSCSCs and MP2 differentiate and gain resistance to NK cell-mediated cytotoxicity, we determined the susceptibility of OSCSCs (Figure 2C) and MP2 (Figure S2A in Supplementary Material) to NK cell-mediated lysis when the tumors were treated with supernatants from IL-2 + anti-CD16mAb + sAJ2-treated NK cells cells with each of IFN-γ and TNF-α antibody alone or with the combination of the two antibodies. The addition of each of anti-TNF-α and anti-IFN-γ alone to IL-2 + anti-CD16mAb + sAJ2 resulted in no or moderate inhibitory effect on the induction of resistance of OSCSCs (Figure 2C) and MP2 (Figure S2A in Supplementary Material) to NK cells. However, the addition of both antibodies together completely restored the sensitivity of OSCSCs (Figure 2C) and MP2 (Figure S2A in Supplementary Material) to untreated and IL-2-activated NK cells. The addition of TNF-α antibody alone to IL-2 + anti-CD16mAb + sAJ2-treated NK cell supernatant was able to prevent upregulation of CD54 surface expression on differentiated OSCSCs (Figure 2D) and MHC-1 on MP2, whereas the effect of anti-IFN-γ alone was more dominant, and it prevented the modulation of CD54, MHC-I, and B7H1 surface expression on both OSCSCs (Figure 2D) and MP2 tumors (Figure S2B in Supplementary Material) and blocked the moderate level of cell death induced by supernatants from IL-2 + anti-CD16mAb + sAJ2-treated NK cells in OSCSCs after differentiation (Figure 2E). Treatment of OSCSCs with supernatants from IL-2 + anti-CD16mAb + sAJ2-treated NK cells in the presence of both TNF-α and IFN-γ antibodies was able to inhibit modulation of surface receptor expression completely (Figure 2D; S2B in Supplementary Material) and prevented cell death (Figure 2E). In addition, OSCSCs differentiated with supernatants from IL-2 + anti-CD16mAb + sAJ2 NK cells demonstrated inhibition of tumor growth when compared to either untreated OSCSCs or those treated with IL-2 + anti-CD16mAb in the absence of sAJ2 (Figure 2F). Treatment of OSCSCs with supernatants from IL-2 + anti-CD16mAb + sAJ2 in the presence of anti-TNF-α had slight enhancing effect on tumor growth, whereas supernatants obtained from anti-IFN-γ in combination with IL-2 + anti-CD16mAb + sAJ2-treated NK cells enhanced tumor cell growth substantially. Supernatants from IL-2 + anti-CD16mAb + sAJ2-treated NK cells in combination with both anti-TNF-α and anti-IFN-γ when added to OSCSCs restored tumor cell growth to the levels which were obtained with untreated OSCSCs (Figure 2F).

Next, we determined whether resistance to NK cell-mediated cytotoxicity correlates with a change in cytokine and chemokine secretion by NK cells in cultures with differentiated OSCSCs (Figures 2G,H) and MP2 (Figures S4A,B in Supplementary Material). OSCSCs treated with IL-2 + anti-CD16mAb-activated NK cell supernatants triggered less IFN-γ secretion by NK cells; however, the extent of inhibition was far less than OSCSCs treated with IL-2 + anti-CD16mAb + sAJ2-treated NK cell supernatants (*P* < 0.05) (Figure 2G). The addition of anti-IFN-γ alone, but not anti-TNF-α alone, to IL-2 + anti-CD16mAb + sAJ2 was able to restore the IFN-γ secretion back to the levels observed with untreated OSCSCs or MP2 (Figure 2G; Figure S4A in Supplementary Material). Supernatants from IL-2 + anti-CD16mAb-treated NK cells or IL-2 + anti-CD16mAb + sAJ2-treated NK cells when added to tumors inhibited IL-8 secretion in differentiated tumors cocultured with NK cells (*P* < 0.05) (Figure 2H). OSCSCs and MP2 differentiated with supernatants obtained from NK cells treated with IL-2 + anti-CD16mAb + sAJ2 in the presence of TNF-α antibody alone had little effect on the restoration of IL-8 secretion (Figure 2H; Figure S4B in Supplementary Material). In contrast, the addition of anti-IFN-γ alone, without anti-TNF-α, restored the IL-8 secretion in tumors treated with IL-2 + anti-CD16mAb + sAJ2 in the presence and absence of their cocultures with the NK cells (Figure 2H; Figure S4B in Supplementary Material).

### The Addition of Supernatants Obtained from IL-2 + Anti-CD16mAb + sAJ2-Treated NK Cells Induced Differentiation and Resistance of Stem Cells of Apical Papilla

Similar results to those of OSCSCs and MP2 tumors were also obtained when non-transformed SCAPs were used to culture with supernatants of NK cells treated with IL-2 + anti-CD16mAb + sAJ2 in the presence or absence of anti-IFN-γ and anti-TNF-α (Figure S5 in Supplementary Material). Treatment of SCAPs with supernatants from NK cells activated with IL-2 + antiCD16mAB + sAJ2 + anti-TNF-α had no or minimal effect on the restoration of sensitivity of SCAPs to NK cell-mediated lysis in comparison to IL-2 + anti-CD16mAB + sAJ2 treatment. (Figure S5A in Supplementary Material). However, treatment of SCAPs with supernatants from IL-2 + anti-CD16mAb + sAJ2 + anti-IFN-γ treated NK cells was able to restore sensitivity of SCAPs to NK cell-mediated lysis moderately, and the combination of anti-TNF-α and anti-IFN-γ when added to IL-2 + anti-CD16mAb + sAJ2-treated NK cell supernatants restored the sensitivity of SCAPs to NK cell-mediated lysis completely (Figure S5A in Supplementary Material). Sensitivity to NK cell lysis correlated with the ability of both anti-TNF-α and anti-IFN-γ to block upregulation of CD54 and MHC-1 (Figure S5B in Supplementary Material). Anti-IFN-γ in the absence of anti-TNF-α was able to decrease surface marker expression (Figure S5B in Supplementary Material). SCAPs differentiated with supernatants from IL-2 + anti-CD16mAb + sAJ2-treated NK cells demonstrated decreased cell growth when compared to either untreated SCAPs or those treated with either untreated NK supernatants or supernatants from IL-2 + anti-CD16mAb-treated NK cells in the absence of sAJ2 (Figure S5C in Supplementary Material). Treatment of SCAPs with supernatants from NK cells treated with IL-2 + anti-CD16mAb + sAJ2 and anti-TNF-α had slight enhancing effect on cell growth, whereas supernatants obtained from anti-IFN-γ in combination with IL-2 + anti-CD16mAb + sAJ2-treated NK cells restored cell growth. Supernatants from IL-2 + anti-CD16mAb + sAJ2-treated NK cells in combination with both anti-TNF-α and anti-IFN-γ when added to SCAPs restored cell growth similar to the levels obtained with untreated SCAPs (Figure S5C in Supplementary Material).

### Resistance to NK Cell-Mediated Lysis Induced by Fixed IL-2 + Anti-CD16mAb + sAJ2-Treated NK Cells Was Mediated by Both IFN-γ and TNF-α and Not Each Cytokine Alone

**Figure 3:**
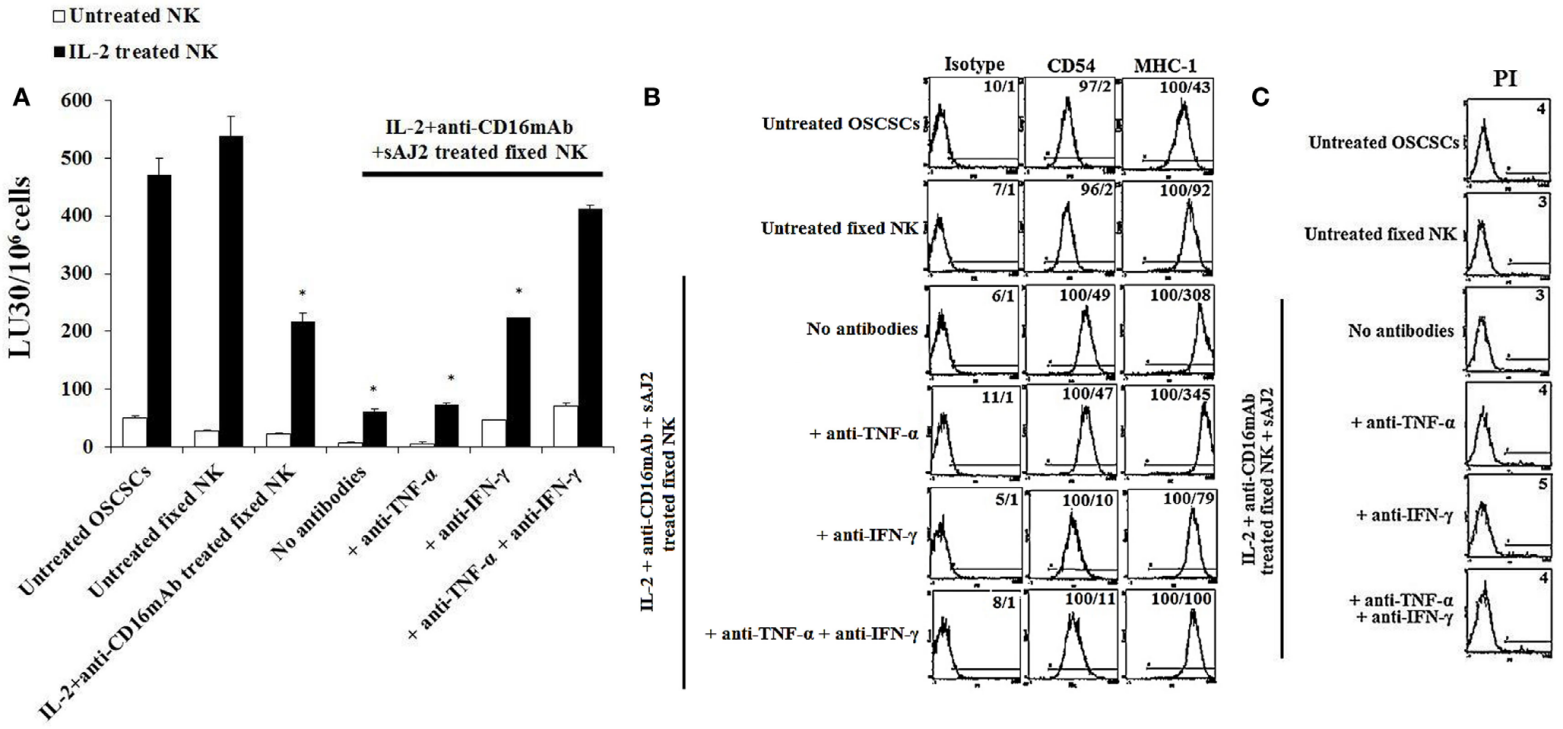
**Paraformaldehyde fixed IL-2 + anti-CD16 mAb + sAJ2-treated NK cells mediated differentiation and resistance of OSCSCs against NK cell-mediated cytotoxicity**. NK cells were left untreated or treated with a combination or IL-2 (1,000 U/mL) and anti-CD16mAb (3 μg/mL) and sAJ2 at 1:3 (NK:sAJ2) ratio in the presence of anti-TNF-α (1:100), anti-IFN-γ (1:100), or combination of anti-TNF-α (1:100) and anti-IFN-γ (1:100) for 18 h. Afterwards, supernatants were removed, and the NK cells were fixed with freshly prepared 2% paraformaldehyde for 15 min. NK cells were then washed three times with 1× PBS and added to tumor cultures. Differentiation of OSCSCs was conducted for 5 days with daily and gradual addition of increasing amounts of fixed NK cells. The complete removal of fixed NK cells from tumor cell cultures prior to the cytotoxicity assays was determined by microscopic assessment. Freshly isolated NK cells were left untreated or treated with IL-2 (1,000 U/mL) for 18 h before the cells were used as effector cells in ^51^Cr release assay against tumor cells cultured with the NK cells. The lytic units 30/10^6^ cells were determined using inverse number of NK cells required to lyse 30% of the target cells ×100. Differences between untreated OSCSCs and those stimulated with fixed IL-2 + anti-CD16mAb-treated NK or IL-2 + anti-CD16mAb-treated NK + sAJ2 with or without anti-TNF-α or anti-IFN-γ but not with the combination of anti-TNF-α and anti-IFN-γ were significant at a *P*-value of <0.05 (*) **(A)**. OSCSCs were treated with fixed NK cells as described in **(A)**. The surface expressions of CD54 and MHC-1 on OSCSCs were assessed after staining with the PE-conjugated antibodies and analyzed using flow cytometry. Isotype control antibodies were used as controls. The numbers on the right hand corner are the percentages and the mean channel fluorescence intensities for each histogram **(B)**. NK cells were prepared as described in **(A)** and added to OSCSCs for 5 days. Afterwards, the tumor cells were washed with 1× PBS, and their viability was determined using PI staining followed by flow cytometric analysis **(C)**. The numbers on the right hand corner are the percentages of dead cells in each histogram.

To determine the effects of cell–cell contact between NK cells and tumor cells on differentiation and resistance of tumor cells to NK cell-mediated cytotoxicity, we cultured purified NK cells untreated or treated with IL-2 + anti-CD16mAb + sAJ2 in the presence or absence of anti-TNF-α and anti-IFN-γ antibodies. Treated NK cells were then washed, fixed with 2% paraformaldehyde and added to OSCSCs and MP2 (Figure 1). Thereafter, the complete removal of fixed NK cells from tumor cell cultures was confirmed by microscopic assessment. Next we determined the susceptibility of OSCSCs and MP2 differentiated with different treatments of fixed NK cells to untreated and IL-2-treated NK cell lysis and the surface expression of CD54 and MHC-1 (Figure 3; Figure S6 in Supplementary Material). Untreated fixed NK cells did not cause any NK cell resistance in OSCSCs (Figure 3A) and MP2 (Figure S6A in Supplementary Material), whereas fixed IL-2 + anti-CD16mAb + sAJ2-treated NK cells induced resistance of OSCSCs (Figure 3A) and MP2 (Figure S6A in Supplementary Material) to both untreated and IL-2-treated NK cell-mediated cytotoxicity (*P* < 0.05). The addition of TNF-α antibody alone had no or minimal inhibitory effect on the resistance of OSCSCs (Figure 3A) and MP2 (Figure S6A in Supplementary Material). Whereas, decreases in OSCSCs and MP2 resistance were observed when fixed NK cells treated with anti-IFN-γ and IL-2 + anti-CD16mAb + sAJ2 were used to differentiate both tumor cell lines. The complete restoration of susceptibility of OSCSCs and MP2 to untreated and IL-2 treated NK cells was observed when the combination of antibodies to IFN-γ and TNF-α and not to each cytokine alone was used with IL-2 + anti-CD16mAb + sAJ2 to treat NK cells before their fixation and addition to tumor cells to induce differentiation (Figure 3A; Figure S6A in Supplementary Material). Resistance of OSCSCs or MP2 induced by fixed NK cells treated with IL-2 + anti-CD16mAb + sAJ2 correlated with the increase in the surface expressions of CD54 and MHC-I on OSCSCs (Figure 3B) and CD54, B7H1, and MHC-1 on MP2 (Figure S6B in Supplementary Material), and the addition of both anti-TNF-α and anti-IFN-γ to IL-2 + anti-CD16mAb + sAJ2-treated NK cells before their use to treat tumor cells inhibited the upregulation of these receptors on tumor cells. The effect of anti-IFN-γ was more potent in the reduction of surface marker expression on tumor cells when compared to anti-TNF-α, since the addition of anti-IFN-γ significantly reduced the increase in surface marker expression (Figure 3B; Figure S6B in Supplementary Material). No or minimal cell death was observed in OSCSCs (Figure 3C) or MP2 (Figure S6C in Supplementary Material) differentiated with IL-2 + anti-CD16mAb + sAJ2-treated and paraformaldehyde-fixed NK cells. Treatment of MP2 with fixed NK cells treated with IL-2 + anti-CD16mAb + sAJ2 + anti-TNF-α had slight enhancing effect on tumor growth, whereas fixed NK cells obtained from anti-IFN-γ in combination with IL-2 + anti-CD16mAb + sAJ2-treated NK cells enhanced tumor cell growth substantially. NK cells treated with IL-2 + anti-CD16mAb + sAJ2 in combination with both anti-TNF-α and anti-IFN-γ when added to MP2 cells restored tumor cell growth to the levels obtained with untreated MP2 cells (Figure S6D in Supplementary Material).

### Monocytes Synergized with NK Cells in the Presence of Bacteria to Induce Differentiation of Tumors

**Figure 4:**
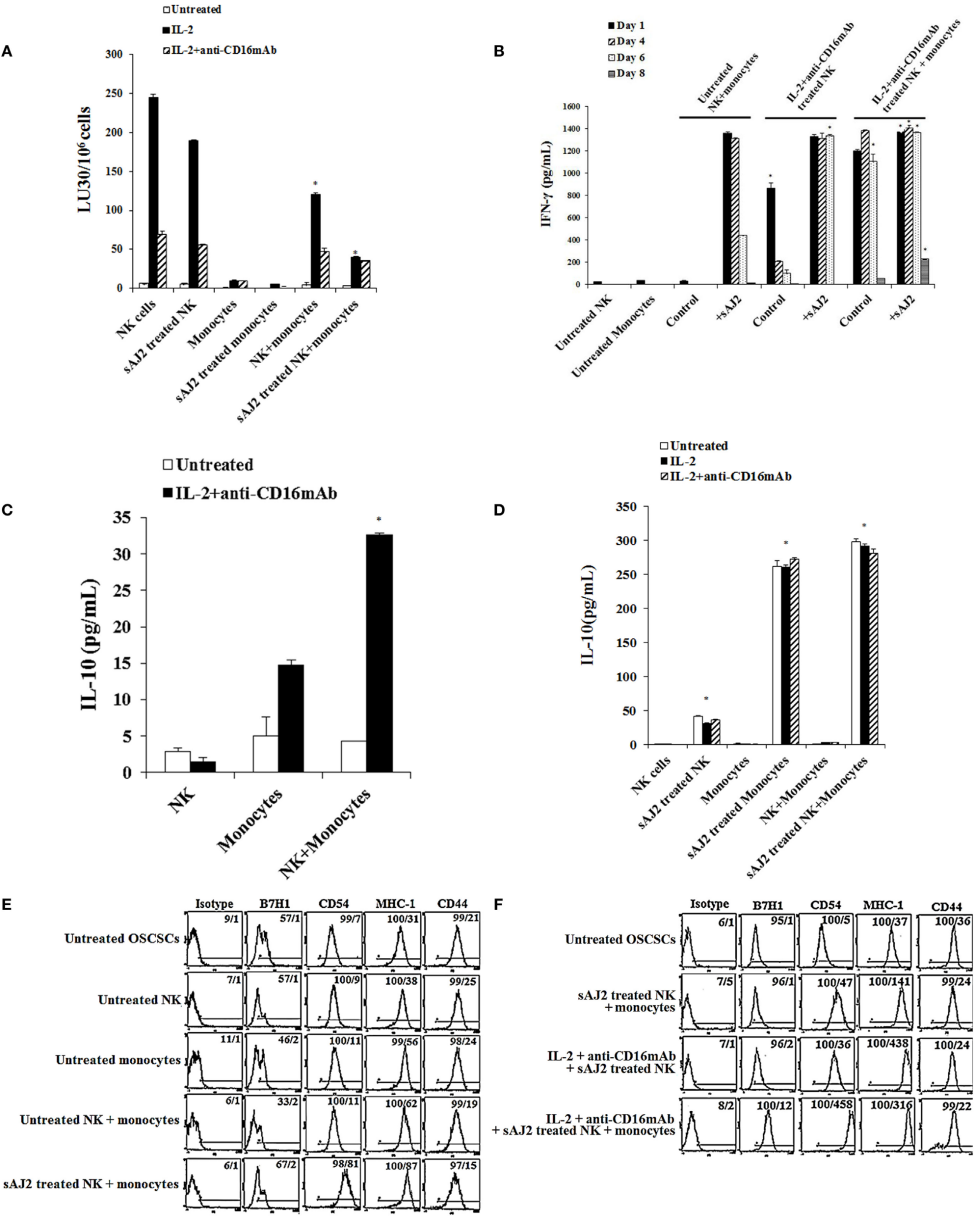
**Monocytes and probiotic bacteria synergistically induce split anergy in NK cells to increase tumor cell differentiation**. Purified NK cells were left untreated, treated with IL-2 (1000U/mL) or a combination of IL-2 (1000U/mL) and anti-CD16mAb (3μg/mL) in the presence or absence of autologous monocytes (1:0.2, NK cell:monocytes) and/or sAJ2 (1:0.5, NK cell:sAJ2) ratios for 18 hours before the supernatants were harvested and added to OSCSCs. Afterwards, untreated OSCSCs and those treated with different NK cell supernatants indicated in the figure were detached from the tissue culture plates, extensively washed with 1X PBS and labeled with ^51^Cr. Freshly isolated NK cells were left untreated or treated with IL-2 (1000U/mL) for 24 hours before the cells were used as effector cells in ^51^Cr release assay against OSCSCs treated with NK supernatants. The lytic units 30/10^6^ cells were determined using inverse number of NK cells required to lyse 30% of the target cells X100. NK cell cytotoxicity was determined using a standard 4 hours ^51^Cr release assay and the lytic units 30/10^6^ were determined using inverse number of NK cells required to lyse 30% of the target cells. Differences between untreated NK cells and those cultured with monocytes and/or sAJ2 were significant at a *P*-value of < 0.05 (*) **(A)**. NK cells were prepared as described in **(A)**. After days 1, 4, 6, and 8, the supernatants were removed from the cocultures, and the levels of IFN-γ secretions were determined using specific ELISAs. Differences between untreated NK cells and those stimulated with IL-2 + anti-CD16mAb-treated NK cells or treated with IL-2 + anti-CD16mAb-treated NK cells + monocytes with or without sAJ2 were significant at a *P*-value of <0.05 (*) **(B)**. Purified NK cells were left untreated or treated with a combination of IL-2 (1,000 U/mL) and anti-CD16mAb (3 μg/mL) in the presence or absence of monocytes (1:0.2, NK cell:monocytes) for 18 h. Afterwards, the supernatants were removed, and the levels of IL-10 were determined using specific ELISAs. Differences between IL-2 + anti-CD16mAb-treated NK cells and IL-2 + anti-CD16mAb-treated NK cells cultured with monocytes were significant at a *P*-value of <0.05 (*) **(C)**. NK cells were prepared as described in **(A)**. After 36 h, the supernatants were removed from the cocultures, and the levels of IL-10 secretions were determined using specific ELISAs. Differences between unstimulated NK cells and those stimulated with sAJ2 and/or monocytes were significant at a *P*-value of <0.05 (*) **(D)**. Purified untreated NK cells were cultured in the presence and absence of untreated monocytes with and without sAJ2 for 18 hours. Then the supernatants were added to OSCSCs for 4 days. Afterwards, OSCSCs were removed and the surface expressions of B7H1, CD54, MHC-1, and CD44 on untreated OSCSCs and those treated with NK cells were assessed after staining with the PE conjugated antibodies and analyzed using flow cytometry **(E)**. NK cells were left untreated or treated with IL-2 (1,000 U/mL) and anti-CD16mAb (3 μg/mL) in the presence of monocytes (1:0.5, NK cell:monocytes) and/or sAJ2 (1:0.5, NK:sAJ2) for 18 h, and then the supernatants were removed and added to OSCSCs for 4 days. Afterwards, OSCSCs were removed, and the surface expressions of B7H1, CD54, MHC-1, and CD44 on untreated OSCSCs and those treated with NK cells were assessed after staining with the PE-conjugated antibodies and analyzed using flow cytometry. Isotype control antibodies were used as controls. The numbers on the right hand corner are the percentages and the mean channel fluorescence intensities for each histogram **(F)**.

To determine the effects of NK cells treated with or without sAJ2 in the presence or absence of monocytes, we assessed cytotoxicity, secretion of IFN-γ and IL-10, and the surface expression of key differentiation markers on OSCSCs after their culture with supernatants from IL-2 + anti-CD16mAb + sAJ2-treated NK cells cocultured with autologous monocytes (Figure 4). As shown in Figure 4A, the highest cytotoxicity was observed when NK cells were treated with IL-2, and the addition of anti-CD16mAb to IL-2-activated NK cells induced split anergy in NK cells. The addition of sAJ2 inhibited moderately the cytotoxic function of both IL-2-treated and IL-2 + anti-CD16mAb-treated NK cells (Figure 4A). As shown previously (2–4, 22) and in this report, the addition of monocytes to NK cells induced split anergy in NK cells which resulted in a significant decrease in NK cell-mediated lysis (Figure 4A). The ability of NK cells to mediate cytotoxicity was drastically lost when NK cells were cultured with both monocytes and sAJ2 (Figure 4A).

To determine whether a drop in cytotoxicity correlated with substantial induction of IFN-γ from NK cells treated with or without IL-2 + anti-CD16mAb in the presence of monocytes and sAJ2, the secretion of IFN-γ were determined at days 1, 4, 6, and 8 post-treatment (Figure 4B). Untreated NK cells cultured with monocytes produced minimal amounts of IFN-γ on the first day, and none was detected on the subsequent days. The addition of sAJ2 to NK cells cocultured with monocytes resulted in a significant increase in IFN-γ production by NK cells, and the levels remained high until day 6 but by day 8 no IFN-γ release was detected (Figure 4B). Without monocytes and sAJ2, IL-2 + anti-CD16mAb-treated NK cells secreted moderate amounts of IFN-γ on day 1, and the levels dropped significantly on day 4 of the co-cultures (Figure 4B). On the other hand, IL-2 + anti-CD16mAb-treated NK cells in the presence of sAJ2 continued to secrete high levels of IFN-γ, and the levels remained continuously high until day 6. IL-2 + anti-CD16mAb-treated NK cells cultured with monocytes also secreted high levels of IFN-γ, and the levels remained high until day 8 (Figure 4B). The longest duration in which NK cells continued to secrete IFN-γ was seen when NK cells were treated with IL-2 + anti-CD16mAb with sAJ2 and cultured with monocytes since at day 8 considerable amounts of IFN-γ were still detectable from the NK cell supernatants (Figure 4B). No increase in cell death above that observed with IL-2 + anti-CD16mAb-treated NK cells was seen when IL-2 + anti-CD16mAb-treated NK cells were cultured with or without monocytes and/or sAJ2 (Figure S7 in Supplementary Material). Next, we determined secretion of IL-10 in cocultures of NK cells with monocytes. NK cells synergized with monocytes to induce IL-10 secretion (Figure 4C), and the levels increased substantially in the presence of sAJ2 (Figure 4D).

Next, we aimed to determine if the increased and persistent production of IFN-γ by IL-2 + anti-CD16mAb-treated NK cells cultured with monocytes and sAJ2 correlated with an increase in differentiation antigens on the surface of OSCSCs (Figures 4E,F). Supernatants obtained from untreated NK cells cultured with sAJ2 and monocytes induced differentiation of OSCSCs as observed by a slight increase in surface expression of B7H1, moderate upregulation of CD54 and MHC-1, and a minor decrease in CD44 on OSCSCs (Figure 4E). Supernatants from IL-2 + anti-CD16mAb-treated NK cells cultured with sAJ2 mediated the next highest level of resistance in OSCSCs and induced on average 7-fold and 11.8-fold increase in surface expression of CD54 and MHC-1 on OSCSCs, respectively (Figure 4F). The combination of IL-2 + anti-CD16mAb + sAJ2-treated NK cells cultured with monocytes was able to synergistically induce significant upregulation of B7H1 (12-fold), CD54 (92-fold), and MHC-1 (8.5-fold) (Figure 4F).

### IL-10 Regulation of NK Cell-Mediated Tumor Differentiation in the Presence of Bacteria and Monocytes Was Much More Pronounced in Untreated NK Cells Than Those Treated with IL-2 and Anti-CD16mAb

**Figure 5:**
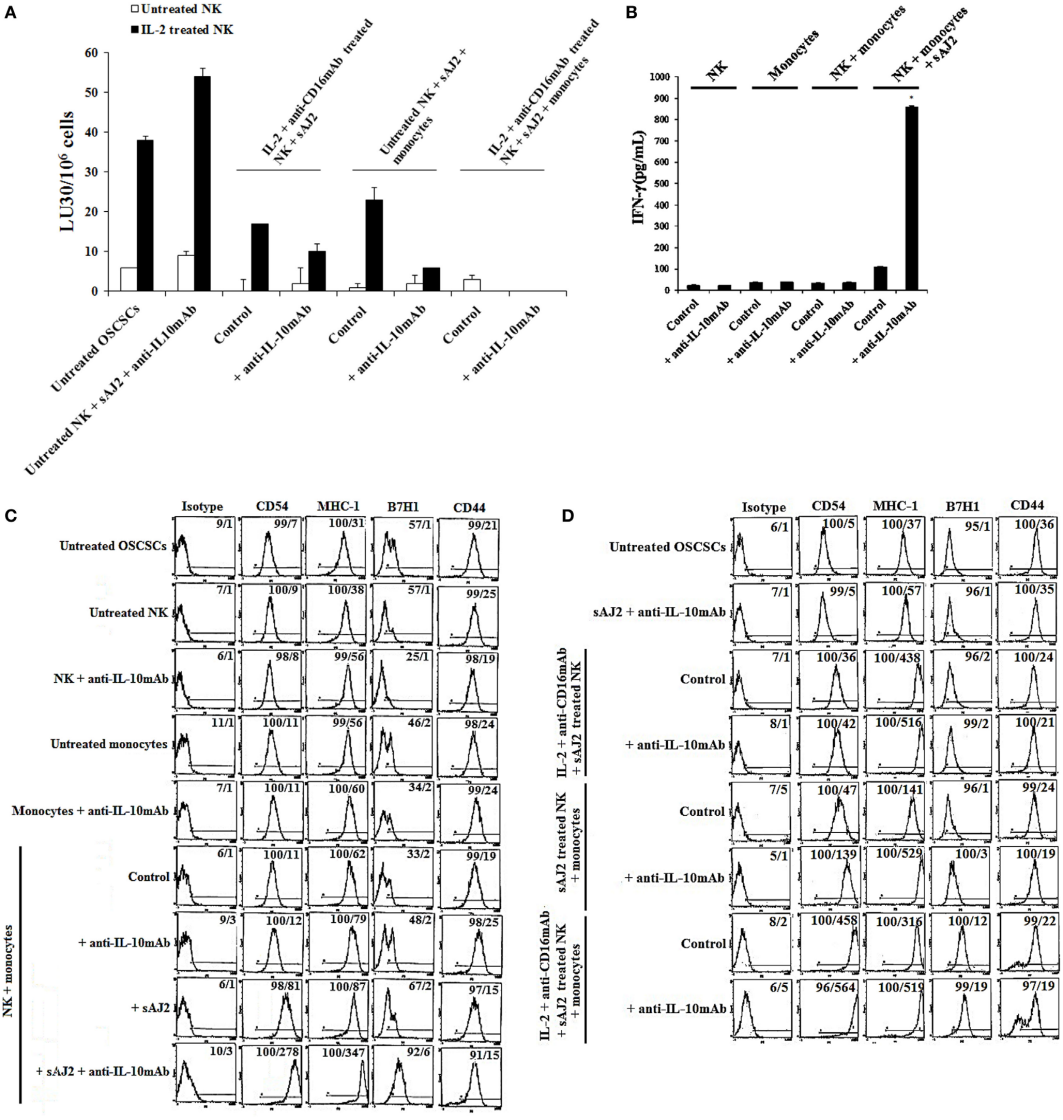

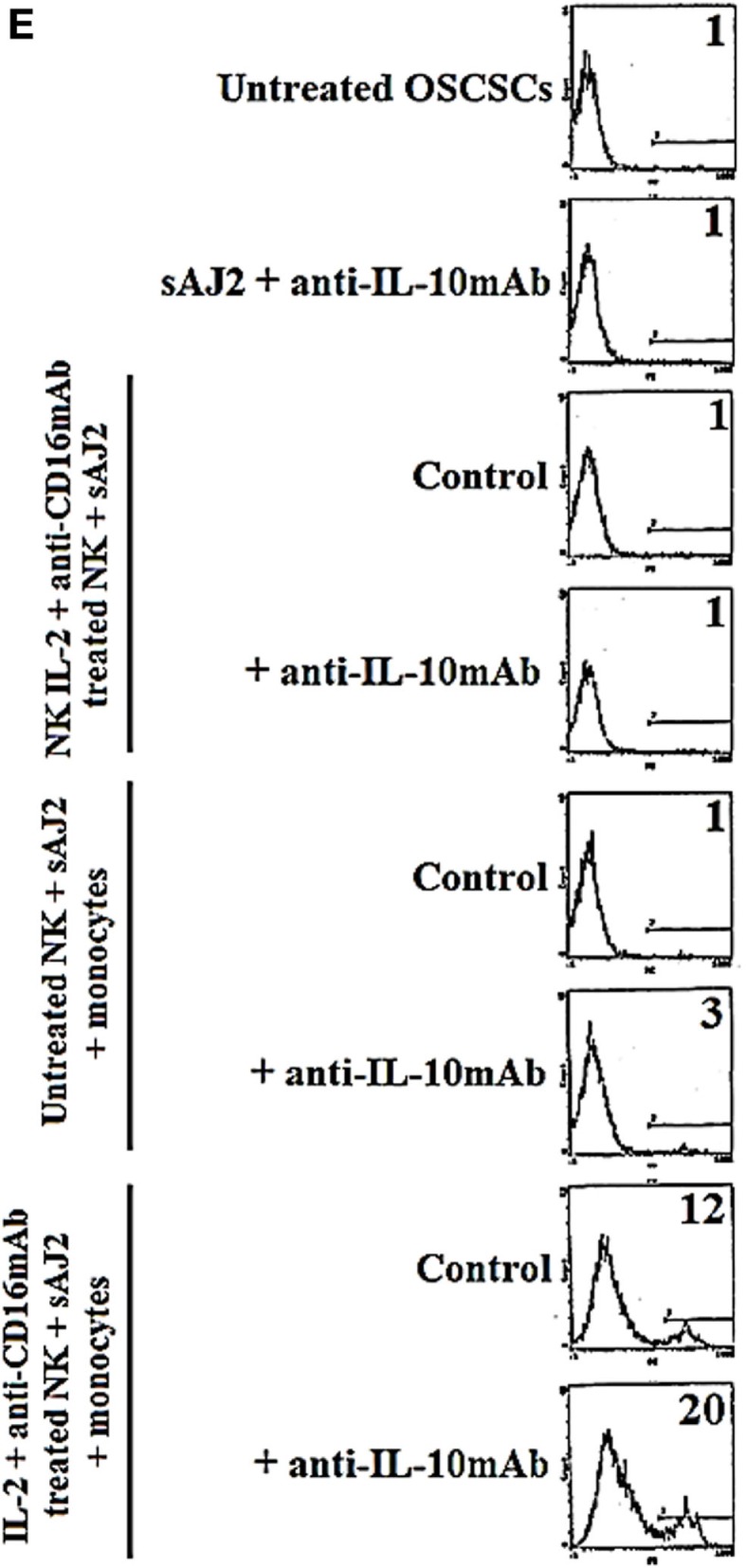
**IL-10 regulation of NK cell mediated CSC differentiation in the presence of sAJ2 and monocytes is much more pronounced in untreated NK cells when compared to those treated with IL-2 and anti-CD16mAb**. Highly purified NK cells were left untreated or treated with IL-2 (1,000 U/mL) and anti-CD16mAb (3 μg/mL) with sAJ2 at 1:3 (NK:sAJ2) ratio, monocytes at 1:1 (NK:monocyte) ratio, and/or anti-IL-10 (10 μg/mL) for 18 h. Afterwards, supernatants were harvested and added to OSCSCs for a period of 3 days. Then, untreated OSCSCs and those treated with NK cell supernatants were detached from the tissue culture plates, washed with 1× PBS and labeled with ^51^Cr. Freshly isolated NK cells were left untreated or treated with IL-2 (1,000 U/mL) for 24 h before the cells were used as effector cells in ^51^Cr release assay against OSCSCs. The lytic units 30/10^6^ cells were determined using inverse number of NK cells required to lyse 30% of the target cells ×100 **(A)**. NK cells were prepared as described in **(A)**, and after an overnight incubation period, the supernatants were harvested, and the levels of IFN-γ secretions were determined using specific ELISAs. Differences between untreated NK cells and those cultured with monocytes and sAJ2 were significant at a *P*-value of <0.05 (*) **(B)**. Untreated NK cells were cultured with autologous monocytes at 1:1 (NK:monocyte) ratio in the presence or absence of anti-IL-10 (10 μg/mL) and/or sAJ2 at 1:3 (NK:sAJ2) ratio for 18 h. Anti-IL-10 (10 μg/mL) was added to untreated NK cells and monocytes and used as controls. After an overnight treatment period, the supernatants were removed and added to OSCSCs for 3 days **(C)**. NK cells supernatants were prepared as described in **(A)** and added to OSCSCs for 3 days **(D)**. Afterwards, untreated and treated OSCSCs were detached from the tissue culture plate and washed with 1× PBS. The surface expression of B7H1, CD54, MHC-1, and CD44 was assessed after staining with the PE-conjugated antibodies and analyzed using flow cytometry. Isotype control antibodies were used as controls. The numbers on the right-hand corner are the percentages, and the mean channel fluorescence intensities for each histogram **(C)** and **(D)**. OSCSCs were differentiated with NK cell supernatants as described in **(D)**, and at the end of the treatment period, the viability of OSCSCs was assessed with propidium iodide staining followed by flow cytometric analysis **(E)**. The numbers on the right hand corner are the percentages of dead cells in each histogram.

Since sAJ2 induce significant IL-10 secretion in untreated NK cells, and the levels substantially decrease when NK cells are activated with IL-2 or IL-2 + anti-CD16mAb (Table S1 in Supplementary Material), we aimed at determining whether such modulation also affects tumor differentiation. To analyze the extent and significance of the regulatory role of IL-10 on untreated and activated NK cell in tumor differentiation, anti-IL-10mAb was added to NK cells in the presence of sAJ2 and/or monocytes, and their supernatants were used in the induction of differentiation, and resistance of OSCSCs to NK cell mediated lysis and secretion of IFN-γ (Figures 5A,B). As shown in Figure 5A, the supernatants obtained from NK cells treated with anti-IL-10mAb + sAJ2 + monocytes induced resistance in OSCSCs. The increase in tumor resistance correlated with the upregulation of CD54, B7H1, and MHC-I on OSCSCs induced by supernatants from NK cells treated with anti-IL-10mAb + sAJ2 + monocytes which contained increased amounts of IFN-γ (Figures 5B,C). Supernatants obtained from the combination of anti-IL-10mAb with IL-2 + anti-CD16mAb-treated NK cells in the presence or absence of monocytes and sAJ2 when added to OSCSCs only moderately increased cell surface receptors when compared to OSCSCs treated with supernatants from NK cells treated with IL-2 + anti-CD16mAb with or without monocytes and sAJ2 in the absence of anti-IL-10mAb (Figure 5D). This correlated with downmodulation of IL-10 secretion when NK cells are activated with IL-2 or IL-2 + anti-CD16mAb in the presence of sAJ2 (Table S1 in Supplementary Material). Supernatants from NK cells treated with IL-2 + anti-CD16mAb-treated NK cells cocultured with sAJ2 and monocytes in the presence of anti-IL-10mAb when added to OSCSCs increased surface receptor expression moderately and induced cell death of OSCSCs when compared to those in the absence of anti-IL-10mAb (Figures 5D,E). Thus, IL-10 appears to be important for the controlled differentiation of the cells, allowing for survivial.

## Discussion

Previous studies have attributed significant health enhancing effect to probiotic bacteria; however, the underlying mechanisms for their effect on NK cells have not been elucidated yet. Here, we demonstrate that probiotic bacteria significantly induce split anergy in NK cells, resulting in substantial secretion of cytokines important in the differentiation of cells and cessation of inflammation. Probiotic bacteria induced significant levels of pro- and anti-inflammatory cytokines, chemokines, and growth factors by NK cells in the absence of other activating signals (Table S1 in Supplementary Material). Interestingly, the addition of probiotic bacteria in the presence of IL-2 or IL-2 + anti-CD16mAb decreased release of the majority of pro- and anti-inflammatory cytokines, chemokines, and growth factors, with the exception of a few, most notably IFN-γ and IL-1Ra which were significantly upregulated. These data indicate the potential balancing effect of bacteria on NK cell activation. On the one hand, probiotic bacteria stimulate NK cells to produce very high secretion of cytokines and chemokines in the absence of other NK cell activators, but on the other hand, they dampen secretion by NK cells when added to those activated with IL-2 or IL-2 + anti-CD16mAb. Such potential balancing effect of the bacteria may be important to retain activation signals at a level that do not result in extremely high or low signaling in NK cells in order to prevent over or under activation of NK cells, respectively. This may likely be one reason why increased cell death in NK cells was not observed above and beyond that which was seen by IL-2 + anti-CD16mAb-treated NK cells in which we had previously reported activation-induced cell death. Indeed, when bacteria were added to IL-2 + anti-CD16mAb-treated NK cells, 55-fold increase in IFN-γ secretion could be seen by certain bacterial species when compared to the same treatment without the addition of the bacteria (Table S1 in Supplementary Material).

Unique cell profiles, in respect to the extent of sensitivity to NK cell-mediated cytotoxicity, cell growth, and viability, were observed when OSCSCs, MP2, and SCAPs were treated with either supernatant or fixed IL-2 + anti-CD16mAb + sAJ2-treated NK cells (Figures 2 and 3; Figures S2 and S5 in Supplementary Material). Furthermore, different amounts of NK supernatants containing IFN-γ were required for the complete differentiation of each of the three cell types. When NK cells were stimulated with IL-2 + anti-CD16mAb and bacteria in the presence of monocytes, which induce IFN-γ and TNF-α at a much higher level than those cultured without monocytes, a substantial increase in the resistance to NK cell-mediated cytotoxicity as well as some increase in cell death of differentiated stem cells could be observed, although the levels differed depending on stem cell type (Figure 4). These results indicated that each stem cell type has a different threshold for undergoing cell death after differentiation even though they all become resistant to NK cell-mediated cytotoxicity, and this is likely due to the expression levels of IFN-γ and TNF-α receptors after differentiation (4, 24, 25). In this regard, we have found OSCSCs to have higher cell death after differentiation with relatively lower amounts of supernatants, whereas MP2 was resistant at higher levels of NK supernatant treatment and did not undergo cell death (4, 24, 25). The observed differences could be the reason why specific tumors are more effectively controlled than others, providing the rationale for designing effective treatment strategies.

Differentiation of OSCSCs and MP2 stem-like tumors by IL-2 + anti-CD16mAb + sAJ2-treated NK cells inhibited greatly the secretion of cytokines and chemokines when they were cultured with freshly isolated IL-2-activated NK cells. Addition of anti-IFN-γ antibody but not anti-TNF-α antibody was able to reverse the inhibition of IFN-γ and IL-8 secretion resulting in the restoration of their secretion. This observation is of great significance since it indicates that cellular differentiation is an important step in the prevention of inflammation. Indeed, IFN-γ secreted by the activated NK cells limits its own production when the stem cells are differentiated which establishes a loop in which the higher the levels of IFN-γ secreted from NK cells the more inhibition of inflammation can be seen. Therefore, any suppression in NK cell-mediated secretion of IFN-γ may cause chronicity of inflammation.

Certain *Lactobacillu*s species and *S. thermophiles* in contrast to *bifidobacteria* induce a Th1-type cytokine profile, i.e., increase in IL-12 and IFN-γ and decrease in IL-10 cytokines whereas *bifidobacteria* triggers relatively more of IL-10 and IL-6 and less of IL-12 and IFN-γ from NK cells which is a Th2-type profile (Table S1 in Supplementary Material). The role of IL-10 in the regulation of IFN-γ secretion has clearly been shown in a number of previous studies; however, its role in the differentiation of the cells has not been shown or proposed previously. In this paper, we demonstrate the significance of IL-10 in regulating NK cell-induced differentiation of the tumor cells. It is clear that NK cells exhibit very low secretion of IL-10 in the absence of bacteria, but when activated with probiotic bacteria they induce significant levels of IL-10, and the amounts synergistically increase in the presence of monocytes. Activation of NK cells with IL-2 or IL-2 + anti-CD16mAb decreases secretion of bacteria induced IL-10, indicating the cross regulation of IFN-γ and IL-10. Addition of anti-IL-10mAb increases IFN-γ significantly when added to the cultures of probiotic bacteria-treated NK cells with monocytes in the absence of NK activation with IL-2 or IL-2 + anti-CD16mAb, which results in substantial increases in B7H1, CD54, and MHC-I surface expression, whereas those that are activated with IL-2 or IL-2 + anti-CD16mAb in the presence of probiotic bacteria only moderately increase surface expression of the above-mentioned receptors in the presence and absence of monocytes after treatment with anti-IL-10mAb. Since IL-2 or IL-2 + anti-CD16mAb inhibits IL-10 secretion triggered by bacteria, the lack of substantial increase in surface receptors when anti-IL-10mAb is added could be due to the limited induction of IL-10. Similarly, higher increase in tumor differentiation with anti-IL10mAb correlates with increased resistance of tumor cells to NK cell-mediated cytotoxicity which closely correlates to the increase in IFN-γ secretion. Therefore, IL-10 is an important regulator of differentiation of the cells and limits over differentiation of the cells which may result in their cell death. Addition of IL-10 to NK cells inhibited IFN-γ secretion (data not shown).

Finally, the *in vivo* relevance to our *in vitro* results was obtained when NK cells expanded by the combination of IL-2 + anti-CD16mAb + sAJ2 injected intravenously to humanized mice were found to increase differentiation of the stem-like/poorly differentiated implanted tumors in the oral cavity and pancreas and resulted in decreased tumor growth, inhibition of tumor metastasis and decrease in inflammation (manuscript submitted). These results demonstrate that tumor differentiation by the NK cells is important in limiting tumor growth, and that probiotic bacteria in combination with NK activating cytokines are able to condition NK cells to promote increased differentiation of the tumors. Differentiation of the tumors with NK cells is not only important to decrease the tumor load, but it is also essential for the activation of tumor infiltrating CD8^+^ T cells to either eliminate the MHC-I-expressing tumor cells or keep the load of the tumors low.

We have previously shown that pathogenic bacteria *Fusobacterium nucleatum* induces significant death of a number of immune effectors via outer membrane proteins Fap2 and RadD potentially limiting the functional ability of immune effectors to lyse tumors or induce effective levels of IFN-γ secretion (31–34). In contrast, we did not observe any cell death contributed either by the sAJ2 bacteria alone or in synergy with other activators of NK cells (Figure S7 in Supplementary Material). This observation is of significance since it may point to the underlying differences between pathogenic and probiotic microorganisms in their mode of action. Indeed, our future studies will focus on determining whether differences between pathogenic and probiotic bacteria in triggering optimal or suboptimal cellular differentiation are responsible for either detrimental or beneficial effects, respectively.

Selection and differentiation of tumors are two important functions mediated by cytotoxic and split anergized NK cells, respectively. Both functions of NK cells play a significant role in prevention of cancer progression. In this paper, we provided evidence that probiotic bacteria signal NK cells to secrete high amounts of IFN-γ and TNF-α, which are substantially more than those induced by IL-2 and CD16 signaling; therefore, they are capable of providing augmented differentiation of the tumors which is important for the inhibition of tumor growth and metastasis and increased effectiveness of chemotherapy (4). In addition, they may be used to alleviate certain inflammatory diseases since regulated differentiation of the cells would likely provide an anti-inflammatory microenvironment leading to the cessation of the inflammation during inflammatory conditions. Indeed, the *in vivo* data obtained with a small cohort of human subjects consuming the probiotic bacteria used in this study demonstrated resolution of chronic mouth ulcers, alleviation of pain and intestinal discomfort, diarrhea and food intolerance, that were accompanied by decrease in the cholesterol level and an increase in the numbers of NK cells (manuscript in preparation).

## Conflict of Interest Statement

The authors declare that the research was conducted in the absence of any commercial or financial relationships that could be construed as a potential conflict of interest.
