# Supplementary material for: Augmented IFN-γ and TNF-α Induced by Probiotic Bacteria in NK Cells Mediate Differentiation of Stem-Like Tumors Leading to Inhibition of Tumor Growth and Reduction in Inflammatory Cytokine Release; Regulation by IL-10
Source: Front Immunol. 2015 Dec 2;6:576. doi: 10.3389/fimmu.2015.00576 (PMC4667036; doi:10.3389/fimmu.2015.00576)
Supplement: Supplementary file 1 [file Data_Sheet_1.DOCX]

**Supplementary Data**

**Supplemental Table Legend**

**Table 1. Production of cytokines, growth factors, and chemokines by NK cells treated with bacterial strains**

Purified NK cells from healthy donors were left untreated or treated with IL-2 (1000 units/ml) or the combination of anti-CD16mAb (3μg/ml) and IL-2 (1000 units/ml) in the presence or absence of probiotic bacteria sAJ2 at 1:5 ratio (NK:sAJ2) for 18 hours. Afterwards, the levels of cytokines, growth factors and chemokines were determined using Bio-Plex Pro Human Cytokine 27-plex Array Kit.

| **Table 1 Cytokines, Growth Factors, and Chemokines** | | | | | | | | | | |  |  |  |  |  |  |  |
| --- | --- | --- | --- | --- | --- | --- | --- | --- | --- | --- | --- | --- | --- | --- | --- | --- | --- |
| **Bacteria** | **Treatments** | **IL-6** | **IFN-γ** | **IL-1ra** | **TNF-α** | **IL-1B** | **IL-10** | **IL-12p70** | **G-CSF** | **IL-8** | **GM-CSF** | **RANTES** | **Eotaxin** | **IP-10** | **IL-13** | **MCP-1** | **IL-17** |
| **Control** | NK | 38 | 34 | 41 | 44 | 4 | 6 | 2 | 28 | 281 | 86 | 2564 | 5 | 64 | 2 | 258 | 33 |
|  | NK+IL-2 | 55 | 99 | 439 | 137 | 10 | 6 | 5 | 54 | 1148 | 100 | 3151 | 12 | 137 | 5 | 525 | 51 |
|  | NK+IL-2+anti-CD16mAb | 39 | 138 | 466 | 166 | 22 | 8 | 7 | 65 | 1480 | 102 | 2536 | 20 | 114 | 5 | 305 | 58 |
| **sAJ2** | NK | 20292 | 222 | 308 | 9634 | 1041 | 200 | 83 | 725 | 12107 | 121 | 1797 | 23 | 40 | 7 | 526 | 54 |
|  | NK+IL-2 | 6034 | 894 | 425 | 2166 | 454 | 38 | 70 | 221 | 2430 | 113 | 1440 | 17 | 34 | 7 | 104 | 50 |
|  | NK+IL-2+anti-CD16mAb | 7944 | 1219 | 618 | 3883 | 839 | 51 | 72 | 350 | 6503 | 201 | 2704 | 35 | 51 | 23 | 107 | 72 |
| **S. thermophilus** | NK | 10222 | 785 | 229 | 18892 | 1381 | 63 | 515 | 149 | 3326 | 131 | 2580 | 40 | 64 | 9 | 502 | 74 |
|  | NK+IL-2 | 1773 | 1188 | 465 | 2413 | 390 | 17 | 132 | 87 | 1341 | 124 | 2197 | 21 | 45 | 9 | 169 | 65 |
|  | NK+IL-2+anti-CD16mAb | 2475 | 2594 | 434 | 4917 | 642 | 23 | 163 | 101 | 1841 | 177 | 2097 | 29 | 58 | 15 | 332 | 57 |
| **B. breve** | NK | 9726 | 99 | 196 | 2904 | 200 | 127 | 10 | 363 | 3975 | 101 | 1662 | 15 | 32 | 5 | 265 | 53 |
|  | NK+IL-2 | 3402 | 284 | 470 | 1868 | 165 | 36 | 21 | 138 | 1912 | 127 | 1988 | 22 | 35 | 9 | 72 | 57 |
|  | NK+IL-2+anti-CD16mAb | 7261 | 844 | 565 | 1498 | 421 | 68 | 26 | 289 | 4929 | 180 | 3934 | 29 | 55 | 28 | 153 | 72 |
| **B. longum** | NK | 60895 | 158 | 312 | 5880 | 813 | 298 | 42 | 938 | 9507 | 131 | 2325 | 32 | 38 | 9 | 80 | 74 |
|  | NK+IL-2 | 7287 | 671 | 472 | 2896 | 408 | 52 | 47 | 244 | 2195 | 135 | 2189 | 33 | 46 | 11 | 33 | 73 |
|  | NK+IL-2+anti-CD16mAb | 2916 | 1282 | 461 | 4240 | 498 | 68 | 33 | 386 | 2906 | 158 | 1088 | 28 | 42 | 19 | 75 | 69 |
| **L. acidophilus** | NK | 10357 | 366 | 181 | 6858 | 819 | 62 | 299 | 141 | 6879 | 110 | 2553 | 16 | 77 | 5 | 400 | 63 |
|  | NK+IL-2 | 2095 | 1329 | 487 | 1151 | 335 | 17 | 136 | 89 | 1116 | 127 | 2353 | 21 | 51 | 8 | 112 | 63 |
|  | NK+IL-2+anti-CD16mAb | 3684 | 2715 | 493 | 5663 | 654 | 24 | 155 | 102 | 2004 | 199 | 3999 | 31 | 69 | 23 | 289 | 73 |
| **L. bulgaricus** | NK | 2518 | 144 | 112 | 868 | 293 | 22 | 25 | 77 | 4501 | 121 | 3804 | 7 | 88 | 9 | 609 | 49 |
|  | NK+IL-2 | 837 | 381 | 449 | 439 | 123 | 12 | 21 | 69 | 1997 | 120 | 4737 | 15 | 78 | 10 | 313 | 55 |
|  | NK+IL-2+anti-CD16mAb | 685 | 306 | 383 | 1064 | 141 | 9 | 5 | 59 | 1819 | 136 | 4062 | 6 | 72 | 8 | 305 | 50 |
| **L. paracasei** | NK | 3029 | 186 | 129 | 3809 | 320 | 70 | 70 | 106 | 3716 | 99 | 1069 | 4 | 53 | 8 | 506 | 40 |
|  | NK+IL-2 | 7285 | 2112 | 551 | 6776 | 523 | 47 | 125 | 138 | 3983 | 157 | 6468 | 27 | 90 | 15 | 337 | 63 |
|  | NK+IL-2+anti-CD16mAb | 6867 | 2145 | 573 | 10128 | 721 | 43 | 123 | 153 | 6385 | 215 | >22609 | 40 | 92 | 27 | 427 | 90 |
| **L. plantarum** | NK | 9500 | 282 | 323 | 13887 | 645 | 85 | 130 | 236 | 17756 | 122 | 22609 | 36 | 140 | 8 | 958 | 78 |
|  | NK+IL-2 | 4212 | 5484 | 500 | 9223 | 466 | 43 | 184 | 129 | 3415 | 181 | 1923 | 36 | 115 | 14 | 427 | 73 |
|  | NK+IL-2+anti-CD16mAb | 3679 | 3087 | 575 | 7942 | 714 | 37 | 155 | 125 | 3247 | 216 | 4147 | 40 | 92 | 20 | 335 | 78 |
| **B. infantis** | NK | 6136 | 275 | 179 | 5728 | 249 | 55 | 66 | 145 | 4265 | 123 | 2234 | 18 | 57 | 1 | 451 | 56 |
|  | NK+IL-2 | 6618 | 3073 | 459 | 5103 | 422 | 41 | 115 | 190 | 5090 | 163 | 3225 | 19 | 113 | 19 | 382 | 72 |
|  | NK+IL-2+anti-CD16mAb | 3927 | 1250 | 434 | 5287 | 365 | 34 | 47 | 151 | 3557 | 178 | 2646 | 32 | 88 | 22 | 360 | 65 |

**Supplemental Figure Legends**

**Fig. S1. No significant differences could be observed in cytotoxic activity of NK cells treated with combination of the bacterial strains AJ2 or individual bacterial strain**

NK cells were prepared as described in supplemental table 1 and then used as effector cells against ^51^Cr labeled OSCSCs. NK cell mediated cytotoxicity was determined using a standard 4 hour ^51^Cr release assay and the lytic units 30/10^6^ cells were determined using inverse number of NK cells required to lyse 30% of OSCSCs X100.

**Fig. S1**

**Fig. S2. Induction of differentiation and resistance to NK cell mediated lysis of MP2 tumors treated with IL-2+anti-CD16mAb+sAJ2 NK cell supernatants is mediated by the combination of NK cell induced IFN-γ and TNF-α and not each cytokine alone**

NK cells were left untreated or treated with sAJ2 alone at 1:3 (NK: sAJ2) ratio or treated with IL-2 (1000 units/mL) and anti-CD16mAb (3μg/mL) and sAJ2 at 1:3 (NK: sAJ2) ratio in the presence of anti-TNF-α (1:100), anti-IFN-γ (1:100) or combination of anti-TNF-α (1:100) and anti-IFN-γ (1:100) as shown in the figure for 18 hours. Afterwards, NK cell supernatants were harvested and added to MP2 tumors for 4 days. Afterwards, untreated tumors and those treated with NK cell supernatants were detached from the tissue culture plates, extensively washed with 1X PBS and labeled with ^51^Cr. Freshly isolated NK cells were left untreated or treated with IL-2 (1000 units/mL) for 24 hours before the cells were used as effector cells in ^51^Cr release assay against tumor cells. The lytic units 30/10^6^ cells were determined using inverse number of NK cells required to lyse 30% of the target cells X100. Differences between untreated MP2 and those treated with supernatants from IL-2+anti-CD16mAb+ sAJ2 stimulated NK cells in the absence or presence of anti-TNF-α or anti-IFN-γ were significant at a p value of <0.05 (*). No significant differences could be obtained between untreated MP2 and those treated with supernatants from NK cells treated with IL-2+anti-CD16mAb+sAJ2 with both anti-TNF-α and anti-IFN-γ antibody **(A)**. MP2 tumors were treated with NK cell supernatants as described in Figs. S2A, and after 4 days of treatment the surface expression of MHC-1 and B7H1 on untreated tumor cells or those treated with NK cell supernatants were assessed after staining with the PE conjugated antibodies and analyzed using flow cytometry. Isotype control antibodies were used as controls. The numbers on the right hand corner are the percentages and the mean channel fluorescence intensities for each histogram **(B)**.

**Fig. S2A**


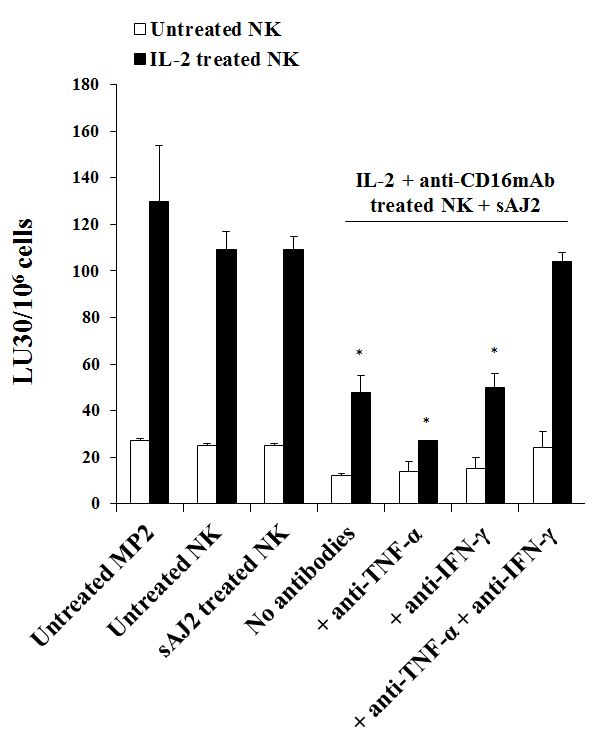


**Fig. S2B**


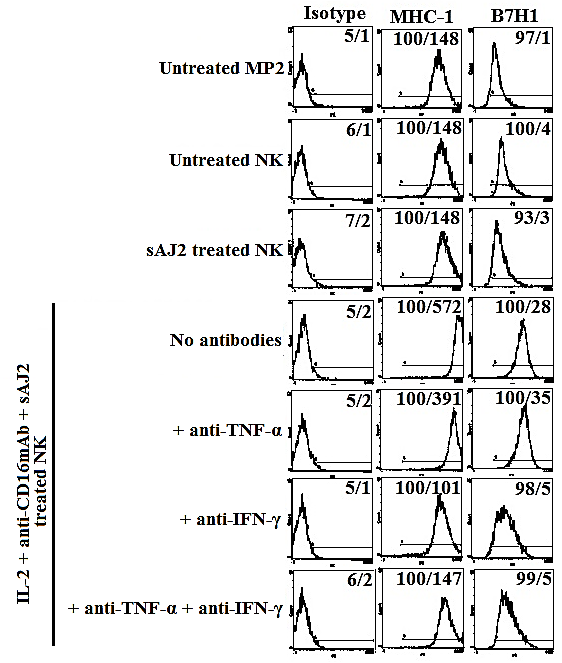


**Fig. S3.** **Treatment with IL-2 or IL-2+anti-CD16mAb in the presence of sAJ2 increased CD107a and CD69 expression on NK cells.**

NK cells were left untreated or treated with IL-2 (1000 units/mL) with and without anti-CD16mAb (3μg/mL) in the presence of sAJ2 at 3:1 (sAJ2: NK) ratio for 18 hours. Afterwards, 5x10^4^ cells from each treatment were stained using PE conjugated antibodies against CD107a **(A)** or CD69 **(B)** and the levels of surface expression were determined by flow cytometric analysis.

**Fig. S3A Fig. S3B**


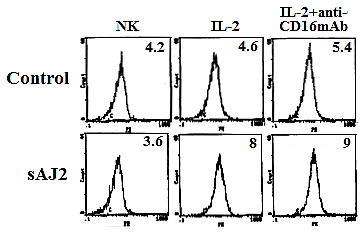

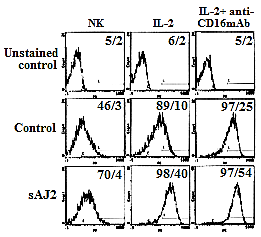


**Fig. S4. MP2 cells cultured with paraformaldehyde fixed IL-2+anti-CD16mAb+sAJ2 treated NK cells triggered less of IFN-γ and IL-8 secretion by NK cells; reversal by anti-IFN-γ antibody**

Purified NK cells were treated with a combination of IL-2 (1000 units/ml) and anti-CD16mAb (3μg/ml) in the presence or absence of sAJ2 at 1:3 ratio (NK:sAJ2) with or without anti-TNF-α (1:100) and/or anti-IFN-γ (1:100) for 18-24 hours. Afterwards, NK cells were fixed with freshly prepared 2% paraformaldehyde for 15 minutes. NK cells were then washed three times with 1X PBS and added to tumor cultures. After 4 days of incubation with NK cells, fixed NK cells were removed from the MP2 tumors, and untreated MP2 tumors and those treated with NK cells were cultured with or without freshly isolated untreated NK cells or NK cells activated with IL-2 (1000 units/ml). After an overnight incubation, the supernatants were removed and the levels of IFN-γ **(A)** and IL-8 **(B)** were analyzed using specific ELISAs.

**Fig. S4A Fig. S4B**


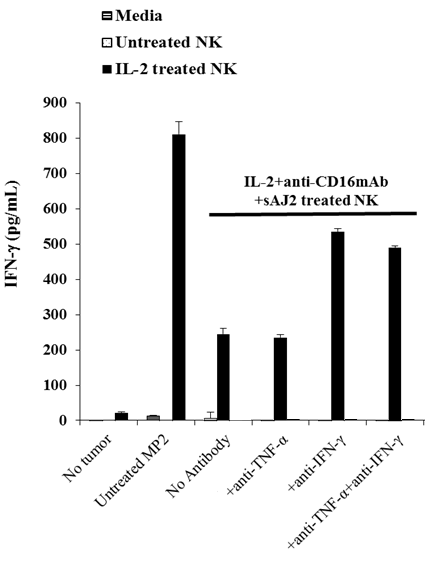

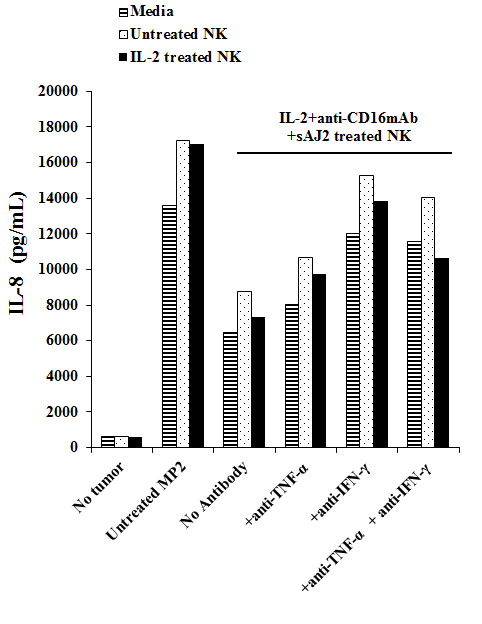


**Fig. S5. Supernatants obtained from IL-2+anti-CD16mAb+sAJ2 treated NK cells induced resistance and differentiation of SCAPs by combination of TNF-α and IFN-γ**

Highly purified NK cells were left untreated or treated with IL-2 (1000 units/mL) and anti-CD16mAb (3μg/mL) with or without sonicated AJ2 (sAJ2) at 1:3 (NK:sAJ2) ratio for 18 hours, after which the NK supernatants were removed and added to SCAPs for a period of 4 days. Thereafter, untreated SCAPs and those treated with NK cell supernatants were detached from the tissue culture plates, washed with 1X PBS and labeled with ^51^Cr. Freshly isolated NK cells were left untreated or treated with IL-2 (1000 units/mL) for 24 hours before the cells were used as effector cells in ^51^Cr release assay against SCAPs. The lytic units 30/10^6^ cells were determined using inverse number of NK cells required to lyse 30% of the target cells X100 Differences between untreated SCAPs and those treated with IL-2+anti-CD16mAb+sAJ2 with or without anti-TNF-α were significant at a p value of <0.05 (*) **(A)**. SCAPs were treated with NK cell supernatants as described in Fig. S5A and the surface expressions of CD54 and MHC-1 on untreated SCAPs and those treated with NK cell supernatants were assessed after staining with the PE conjugated antibodies and analyzed using flow cytometry. Isotype control antibodies were used as controls. The numbers on the right hand corner are the percentages and the mean channel fluorescence intensities for each histogram **(B)**. NK cells were treated as described in Fig. S5A, and supernatants were removed and used for the treatment of SCAPs.  SCAPs at 3X10^5^ cells per well were treated with supernatants from the NK cells for 4 days.  At the end of the incubation, SCAPs were detached and the numbers of cells were assessed using microscopy **(C)**.

**Fig. S5A**
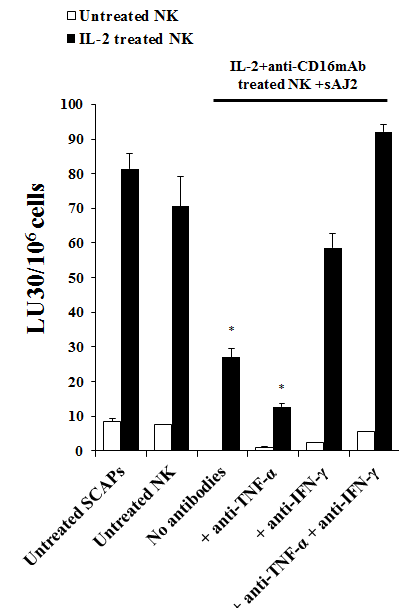


**Fig. S5B**


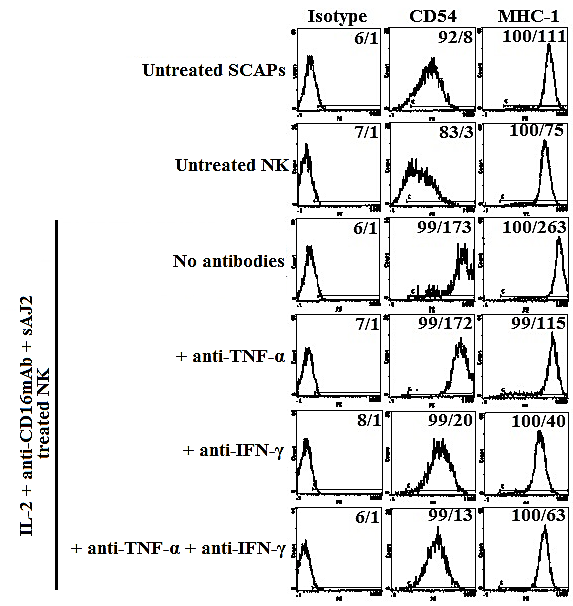


**Fig. S5C**


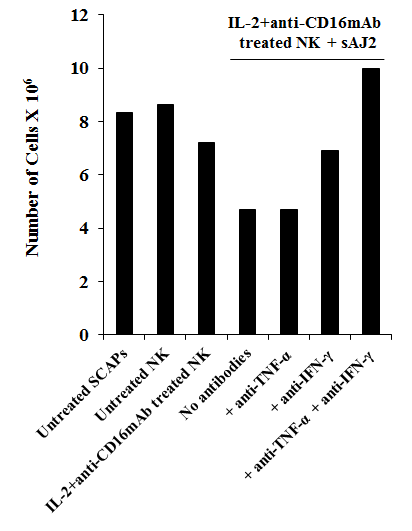


**Fig. S6 Paraformaldehyde fixed IL-2+anti-CD16mAb+sAJ2 treated NK cells mediated differentiation and resistance of MP2 tumors against NK cell mediated lysis**

NK cells were left untreated or treated with a combination or IL-2 (1000 units/mL) and anti-CD16mAb (3μg/mL) and sAJ2 at 1:3 (NK:sAJ2) ratio in the presence of anti-TNF-α (1:100), anti-IFN-γ (1:100) or combination of anti-TNF-α (1:100) and anti-IFN-γ (1:100) for 18 hours. Afterwards, NK cells were fixed with freshly prepared 2% paraformaldehyde for 15 minutes. NK cells were then washed three times with 1X PBS and added to tumor cultures. Differentiation of MP2 cells were conducted for 4 days with daily and gradual addition of increasing amounts of fixed NK cells. The complete removal of fixed NK cells from tumor cell cultures prior to the cytotoxicity assays was determined by microscopic assessment. Freshly isolated NK cells were left untreated or treated with IL-2 (1000 units/mL) for 18 hours before the cells were used as effector cells in ^51^Cr release assay against tumor cells cultured with the NK cells. The lytic units 30/10^6^ cells were determined using inverse number of NK cells required to lyse 30% of the target cells X100. Differences between untreated MP2 and those cultured with fixed IL-2+anti-CD16mAb+sAJ2 treated NK cells with or without anti-TNF-α or anti-IFN-γ but not with the combination of anti-TNF-α and anti-IFN-γ were significant at a p value of <0.05 (*) **(A)**. MP2 tumors were treated with fixed NK cells as described in Fig. S6A. The surface expressions of CD54, B7H1, and MHC-1 on MP2 tumors were assessed after staining with the PE conjugated antibodies and analyzed using flow cytometry. Isotype control antibodies were used as controls. The numbers on the right hand corners are the percentages and the mean channel fluorescence intensities for each histogram **(B)**. NK cells were prepared as described in Fig. S6A and added to MP2 for 4 days. Afterwards, the tumor cells were washed with 1X PBS and their viability was determined using PI staining followed by flow cytometric analysis. The numbers on the right hand corner are the percentages of dead cell in each histogram **(C)**. NK cells were treated as described in Fig. S6A, after which the NK cells were fixed with freshly prepared 2% paraformaldehyde for 15 minutes. NK cells were then washed three times with 1X PBS and added to MP2 cells. MP2 cells were cultured overnight at 3X10^5^ cells per well before they were treated with fixed NK cells for 4 days.   At the end of the incubation, MP2 cells were detached and the numbers of cells were assessed using microscopy **(D)**.

**Fig. S6A**


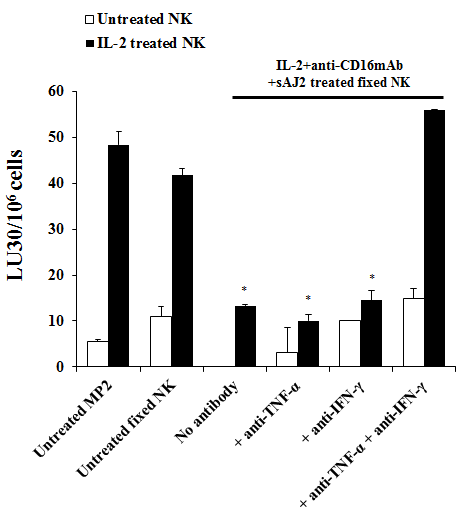


**Fig. S6B**


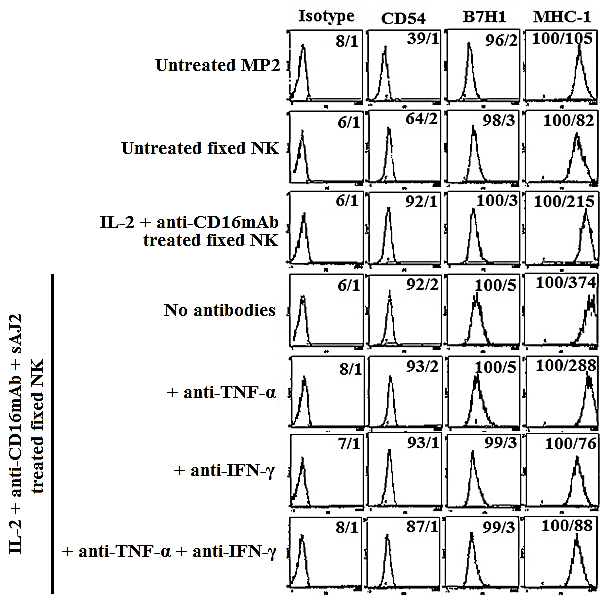


**Fig. S6C Fig. S6D**


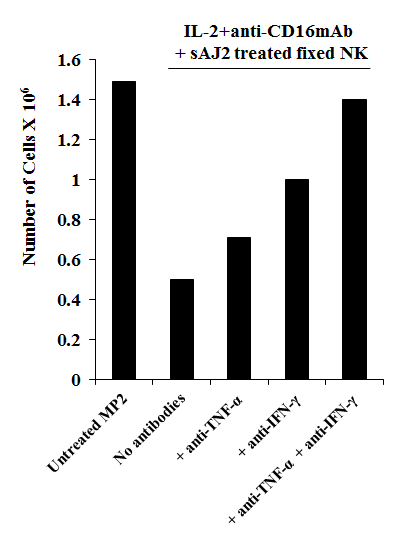

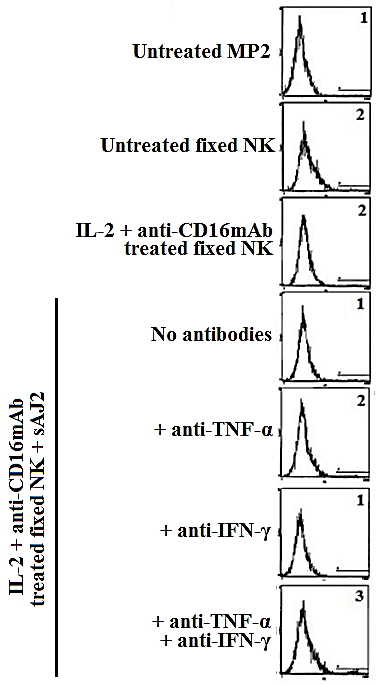


**Fig. S7.** **sAJ2 did not induce cell death in NK cells**

Purified NK cells were left untreated or treated with IL-2 (1000 units/ml) or a combination of IL-2 (1000 units/ml) and anti-CD16mAb (3μg/ml) in the presence or absence of autologous monocytes (1:0.2, NK:monocytes) and/or sAJ2 (1:3, NK:sAJ2) for 24 hours. Afterwards, the viability of untreated and treated NK cells was assessed using PI staining followed by flow cytometric analysis.

**Fig. S7**

**
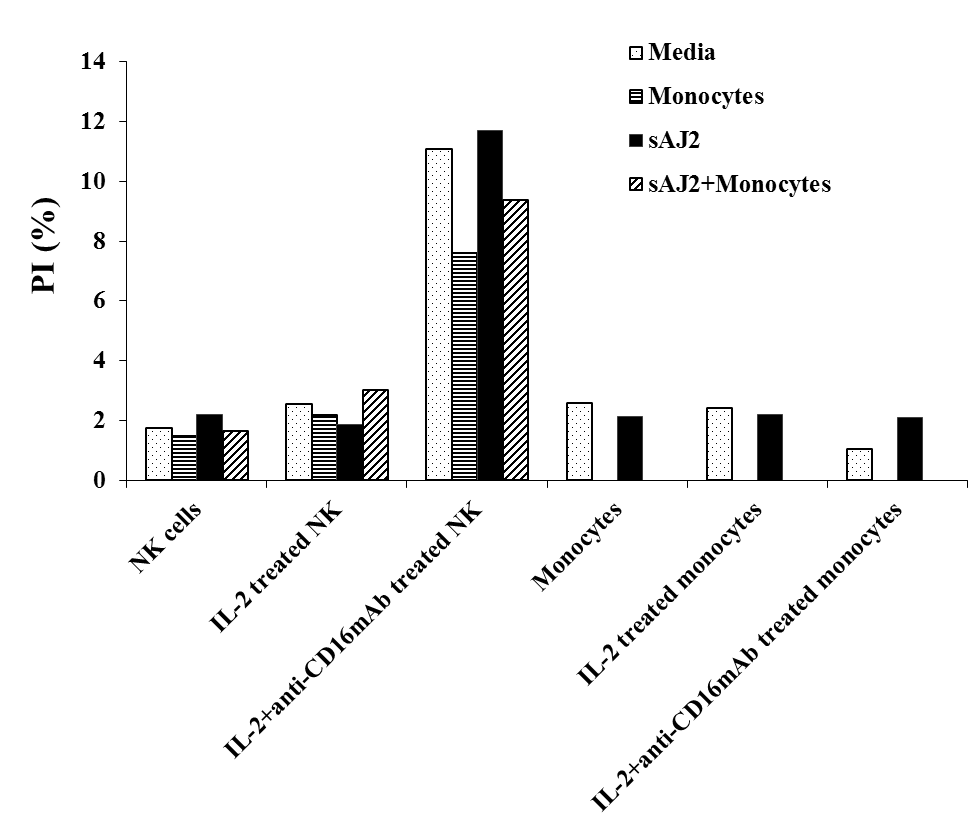
**
